# Synthetic hydrophobic peptides derived from MgtR weaken *Salmonella* pathogenicity and work with a different mode of action than endogenously produced peptides

**DOI:** 10.1038/s41598-019-51760-2

**Published:** 2019-10-24

**Authors:** Mariana Rosas Olvera, Preeti Garai, Grégoire Mongin, Eric Vivès, Laila Gannoun-Zaki, Anne-Béatrice Blanc-Potard

**Affiliations:** 1 Laboratoire de Dynamique des Interactions Membranaires Normales et Pathologiques, Université Montpellier, case 107, Place Eugène Bataillon, 34095 Montpellier cedex 5, France; 2 CNRS, UMR5235, 34095 Montpellier Cedex 05, France; 3 CRBM, CNRS UMR 5237, 1919 route de Mende, 34293 Montpellier, France

**Keywords:** Antibiotics, Infection, Bacteriology

## Abstract

Due to the antibiotic resistance crisis, novel therapeutic strategies need to be developed against bacterial pathogens. Hydrophobic bacterial peptides (small proteins under 50 amino acids) have emerged as regulatory molecules that can interact with bacterial membrane proteins to modulate their activity and/or stability. Among them, the *Salmonella* MgtR peptide promotes the degradation of MgtC, a virulence factor involved in *Salmonella* intramacrophage replication, thus providing the basis for an antivirulence strategy. We demonstrate here that endogenous overproduction of MgtR reduced *Salmonella* replication inside macrophages and lowered MgtC protein level, whereas a peptide variant of MgtR (MgtR-S17I), which does not interact with MgtC, had no effect. We then used synthetic peptides to evaluate their action upon exogenous addition. Unexpectedly, upon addition of synthetic peptides, both MgtR and its variant MgtR-S17I reduced *Salmonella* intramacrophage replication and lowered MgtC and MgtB protein levels, suggesting a different mechanism of action of exogenously added peptides versus endogenously produced peptides. The synthetic peptides did not act by reducing bacterial viability. We next tested their effect on various recombinant proteins produced in *Escherichia coli* and showed that the level of several inner membrane proteins was strongly reduced upon addition of both peptides, whereas cytoplasmic or outer membrane proteins remained unaffected. Moreover, the α-helical structure of synthetic MgtR is important for its biological activity, whereas helix-helix interacting motif is dispensable. Cumulatively, these results provide perspectives for new antivirulence strategies with the use of peptides that act by reducing the level of inner membrane proteins, including virulence factors.

## Introduction

Recent studies based on genomics and natural product discovery have led to a large increase in the number and diversity of small proteins (size below 50 amino-acids, hereafter called peptides) identified to be produced by bacteria^1–3^. An important proportion of newly identified peptides are located in the bacterial membrane^4,5^. Among biologically active peptides, hydrophobic bacterial peptides harboring a single transmembrane domain have emerged as regulatory molecules that can interfere with bacterial membrane proteins, such as histidine kinases or transporters, thereby modulating protein partner’s activity and/or stability^6–12^. Of interest, MgtR is a hydrophobic peptide of 30 amino-acids identified in *Salmonella enterica* serovar Typhimurium (*S*. Typhimurium), encoded by the *mgtCBR* operon, that promotes the degradation of the MgtC virulence factor by FtsH protease, but has no effect on the stability of MgtB magnesium transporter that is expressed from the same operon^6,13^. MgtR has been shown to directly interact with the *Salmonella* MgtC membrane protein and a helix-helix interaction motif (Ala-coil, characterized by three small residues in heptad repeat) has been implicated in this interaction^6^. Disruption of the Ala-coil motif in MgtR upon introduction of a large hydrophobic residue (S17I) did neither prevent expression nor membrane location of MgtR, but abrogated interaction with MgtC^6^. As predicted, based on hydrophobicity profile, NMR spectra confirmed that the MgtR peptide has an α-helical structure^14^.

In the context of the antibiotic resistance crisis, antivirulence strategies have emerged as attractive novel therapeutic approaches^15,16^. The MgtC virulence factor has been proposed as target for antivirulence strategies because it is conserved in several bacterial pathogens^17,18^. The function of MgtC, first described in *S*. Typhimurium, is critical for the intramacrophage survival of various unrelated intracellular pathogens (*Salmonella* spp., *Mycobacterium tuberculosis*, *Brucella suis* and *Burkholderia cenocepacia*), as well as so-called extracellular pathogens that can transiently reside within cells (*Yersinia pestis*, *Pseudomonas aeruginosa*)^19–24^. In agreement with its role inside macrophages, MgtC has been shown to be specifically expressed when bacteria reside in macrophages^17,24^. In *Salmonella*, MgtC promotes pathogenicity through a pleiotropic action^25^ that includes inhibition of the *Salmonella*’s own F_1_F_o_ ATP synthase, inhibition of cellulose production and stabilization of PhoP regulatory protein^26–28^.

Overproduction of MgtR reduces intramacrophage survival of a wild-type *S*. Typhimurium strain (14028s), thus indicating that MgtR can lower *Salmonella* virulence^6^. This intracellular growth defect was not linked to a reduced bacterial growth rate. MgtR membrane peptide from *Salmonella* can also interact with MgtC proteins from *Mycobacterium tuberculosis* and *Pseudomonas aeruginosa*^24,29^, whose N-terminal membrane domain (amino-acids 1-130) exhibits 60% identity with the one of *S*. Typhimurium MgtC. Analysis of heterodimer formation between MgtR and the transmembrane helix 4 of *M*. *tuberculosis* MgtC led to a structural model for interaction^14^. *M*. *tuberculosis* and *P*. *aeruginosa* lack *mgtR* gene in their genome, but ectopic expression of *Salmonella mgtR* in these bacteria mimicked the phenotypes reported for *mgtC* deletion mutants^24,29^. Thus, these results showed an antivirulence action of MgtR upon endogenous production in various bacterial pathogens.

The therapeutic interest in peptide modulators of protein-protein interactions in membrane has increased recently^30^. With this context, the aim of the present study is to investigate the antivirulence effect and biological activity of a synthetic MgtR peptide added exogenously to *Salmonella*. We showed that addition of synthetic MgtR peptide to wild-type *Salmonella* reduced its ability to replicate in macrophages and lowered the amount of MgtC protein. Unexpectedly, an MgtR variant mutated in the Ala-coil motif (MgtR-S17I) displayed a similar effect, which contrasted with results obtained with endogenously produced peptides. We demonstrated that addition of both peptides to bacteria impact the stability of several inner membrane proteins. Taken together, our results support an antivirulence effect of MgtR-derived synthetic peptides against *Salmonella*, through an action independent of the Ala-coil motif, which lowers levels of inner membrane proteins essential for virulence.

## Results and Discussion

### Endogenous overproduction of MgtR, but not MgtR-S17I, reduced intramacrophage replication of wild-type *Salmonella* and lowered MgtC protein level

**Figure 1:**
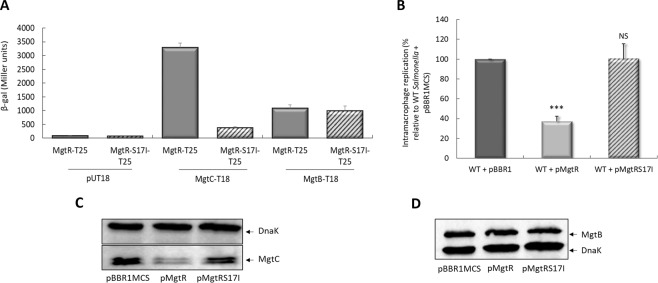
Effect of endogenous production of MgtR and MgtR-S17I on *S*. Typhimurium intramacrophage replication and MgtC and MgtB protein levels. (**A**) *In vivo* protein interaction of endogenous MgtR and MgtR-S17I with MgtC and MgtB in the BACTH system. *E*. *coli* BTH101 strain was co-transformed with plasmids encoding MgtR-T25 or MgtR-S17I-T25 fusion proteins and MgtC-T18 or MgtB-T18, respectively. The basal level of β-galactosidase activity measured with the pUT18 vector is approximately 60 Miller units. Liquid β-galactosidase assays were performed in at least three independent experiments and mean values ± standard errors (SE) are represented. (**B**) Effect of endogenously produced peptides on intramacrophage replication in J774 macrophages. The replication after 18 h of infection of wild-type *Salmonella* 14028s strain carrying pBBR1MCS, pMgtR or pMgtRS17I plasmid was determined by CFU counts. Values presented are the percentage relative to bacteria containing the empty vector pBBR1MCS. Error bars correspond to SE from three independent experiments. *P* values were determined by the Student’s *t* test (***P < 0.001, NS non significant). (**C,D**) Effect of endogenously expressed peptides on the level of MgtC (**C**) and MgtB (**D**) proteins. Western Blot analysis was carried out on lysates of *Salmonella* Δ*mgtR* strain carrying pBBR1MCS, pMgtR or pMgtRS17I plasmid grown in NCE medium supplemented with 10 µM Mg^2+^. Membrane was blotted with anti-MgtC, anti-MgtB and anti-DnaK antibodies. Images are representative of the three independent experiments.

The MgtR peptide harbors an Ala-coil motif (A10, S17, A24) implicated in its interaction with MgtC. An MgtR variant carrying an S17I mutation, where a small residue of the Ala-coil motif is substituted by a large hydrophobic residue, was found to prevent the strong interaction detected between MgtC and MgtR using the bacterial adenylate cyclase two-hybrid (BACTH) system^6^. Accordingly, the high level of β-galactosidase activity observed with MgtC-T18 and MgtR-T25 plasmids, indicative of a robust interaction between MgtC and MgtR, was strongly reduced upon disruption of the Ala-coil motif by the S17I mutation (Fig. 1A). We also investigated the interaction of both peptides with the MgtB transporter (Fig. 1A). The level of β-galactosidase activity measured for MgtB-T18 and MgtR-T25 was much lower than that measured for MgtC-T18, indicating a mild interaction. Moreover, in contrast to MgtC, the level of interaction with MgtR-S17I peptide is similar to that with MgtR, suggesting that the interaction between MgtB and MgtR does not rely on the Ala-coil motif present in MgtR.

We previously showed that the overproduction of MgtR in *S*. Typhimurium wild-type strain significantly reduced its intramacrophage survival^6^, and here we investigated the effect of the MgtR-S17I variant. Wild-type *Salmonella* strain was transformed with a plasmid producing MgtR (pMgtR) or a plasmid producing MgtR-S17I (pMgtRS17I). The survival of both strains was examined in J774 macrophages and compared to that of a strain carrying the pBBR1MCS plasmid vector (Fig. 1B). A significant reduction in the number of intracellular bacteria, 18 h after infection, was found for the strain harboring pMgtR as compared to the strain harboring the empty vector, which is consistent with previous findings^6^. However, no reduction was observed with strain harboring pMgtRS17I. We next monitored the effect of endogenous MgtR and MgtR-S17I peptides on the stability of MgtC virulence factor. For this purpose, we used bacterial cultures of a *Salmonella* strain lacking chromosomal *mgtR* gene (NM516)^6^ to facilitate MgtC detection in a strain harboring the empty vector. Western blot analysis using anti-MgtC antibodies (Fig. 1C) showed that the level of MgtC protein is decreased in the strain carrying pMgtR, but not pMgtRS17I, which corroborates macrophage infection patterns. On the other hand, the level of MgtB protein, monitored using anti-MgtB antibodies, remained unchanged upon expression of both *mgtR* and *mgtR-*S17I (Fig. 1D).

Taken together, these results support that the endogenous overproduction of MgtR led to the degradation of the virulence protein MgtC and thereby reduced the intramacrophage survival. On the other hand, MgtR-S17I had no effect on intramacrophage survival and did not target MgtC to degradation due to the lack of the interacting Ala-coil motif. To further explore the antivirulence potential of MgtR, we carried out experiments with a chemically synthesized peptide that was added exogenously to bacteria.

### Visualization of association of synthetic MgtR peptide with *Salmonella*

**Figure 2:**
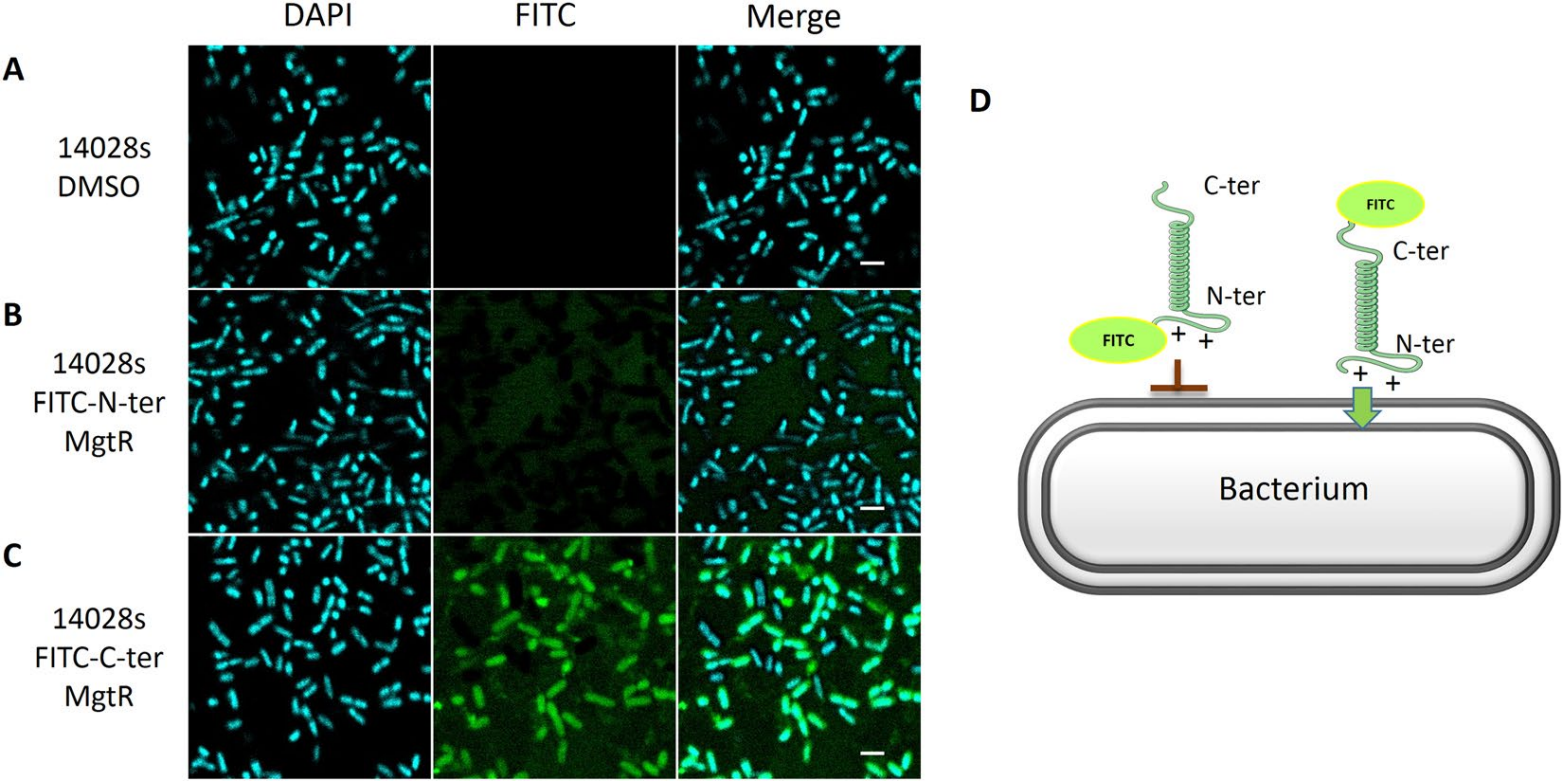
Confocal laser-scanning microscopy of *Salmonella* treated with FITC-labeled MgtR peptide. *S*. Typhimurium 14028s was cultured in LB medium and treated with 120 µM of the FITC-labeled synthetic peptides, where FITC was added at the N-ter (**B**) or C-ter (**C**) end of MgtR for 1 h at 37 °C. As control, DMSO treated bacteria were visualized (**A**). Scale bar is 2 µm. (**D**) Model for the association of MgtR synthetic peptide with the bacteria. The mode of association between peptide and bacteria is unknown but it may involve the interaction of N terminal positively charged residues with bacterial membrane, which could be hindered by N-terminal FITC.

We first investigated the interaction of the synthetic MgtR peptide with *Salmonella* in liquid culture. MgtR labeled with fluorescein isothiocyanate (FITC) dye at its N-terminus or C-terminus was synthesized (see Materials and Methods). After incubation with labeled peptide, bacteria were washed to remove free peptide and imaged by confocal laser scanning microscopy (Fig. 2). Although no association with *Salmonella* was detected with N-terminal FITC-labeled peptide (Fig. 2B), fluorescent *Salmonella* were clearly detected with C-terminal FITC-labeled peptide (Fig. 2C). This result indicates that the synthetic peptide can associate with bacteria and is suitable for biological activity tests.

The endogenous MgtR peptide is predicted to be inserted in bacterial membrane in an N_in_ C_out_ orientation, due to the presence of two positively charged amino-acids in the N-terminus of the peptide. This topological model is supported by bacterial two-hybrid experiments carried out with T25-MgtR fusion protein^6^. Interestingly, the absence of fluorescent bacteria after addition of a synthetic peptide with N-terminal FITC labelling suggests that FITC may hinder the interaction of positively charged N-terminal end with the negatively charged bacterial membrane, thus preventing the association of the peptide with the bacteria (Fig. 2D).

### Exogenous addition of synthetic peptides MgtR and MgtR-S17I reduced intramacrophage replication of wild-type *Salmonella* and lowered levels of MgtC and MgtB

**Figure 3:**
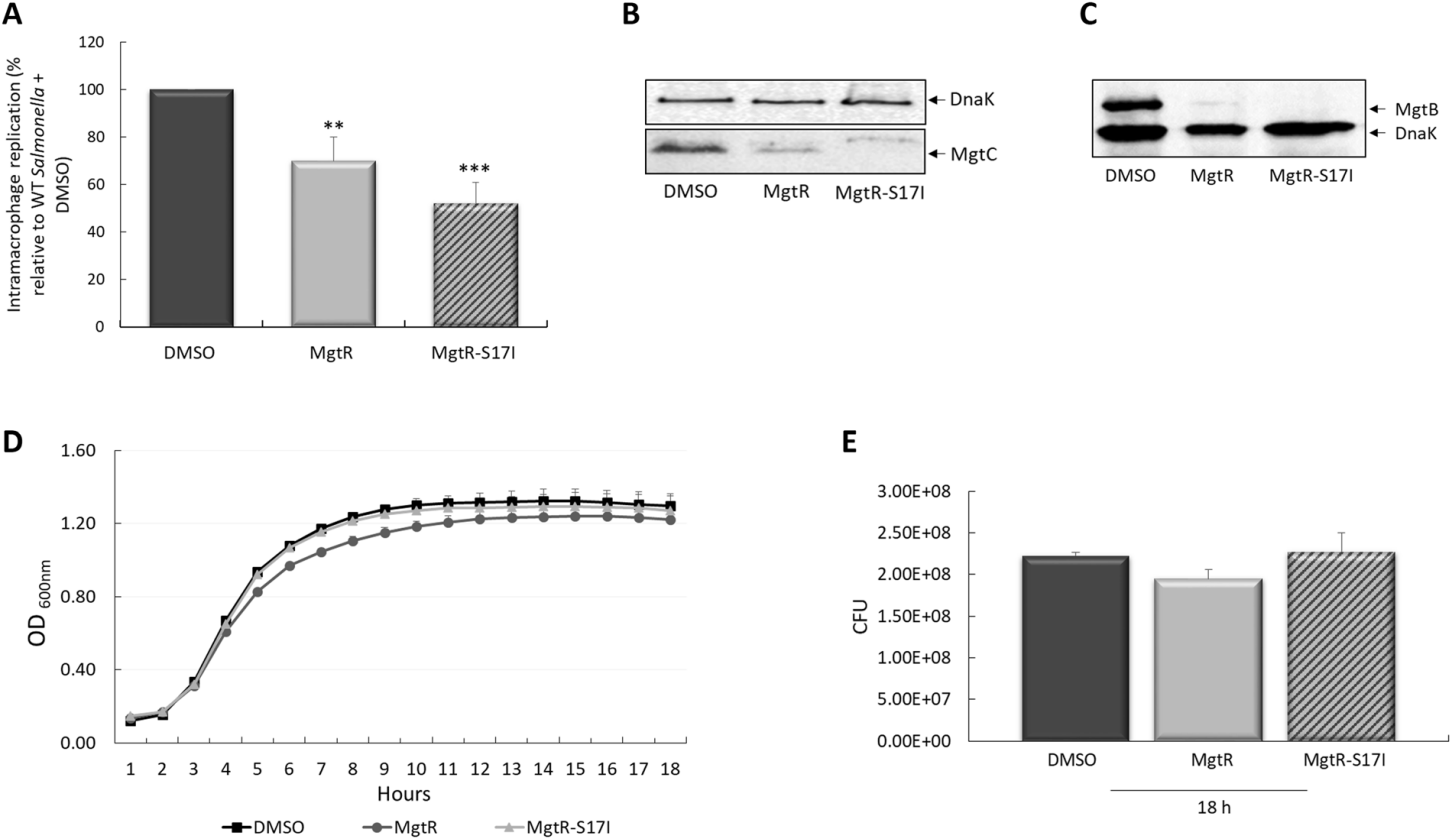
Effect of synthetic MgtR and MgtR-S17I peptides on *S*. Typhimurium intramacrophage replication, bacterial growth and MgtC and MgtB protein levels. (**A**) J774 macrophages were infected with wild-type *S*. Typhimurium 14028s strain. Before infection, bacteria were pretreated with synthetic peptides MgtR or MgtR-S17I (120 µM in DMSO 50% v/v). Pretreatment with the same volume of DMSO 50% v/v is used as control. Intracellular survival was determined 18 h post-infection by counting CFUs and plotting the values as the percentage relative to wild-type bacteria pretreated with DMSO. Data were obtained from three independent experiments and are shown as mean ± SE. Statistical significance was calculated relatively to DMSO treated WT bacteria using Student’s *t* test (***P < 0.001, **P < 0.01). (**B**,**C**) Effect of synthetic peptides on the level of MgtC (B) and MgtB (C) proteins. Western Blot analysis was carried out on lysates of *Salmonella* Δ*mgtR* strain grown in NCE medium supplemented with 10 µM Mg^2+^ and treated with DMSO, MgtR or MgtR-S17I. Membrane was blotted with anti-MgtC, anti-MgtB and anti-DnaK antibodies. Images are representative of three independent experiments. (**D**) Bacterial growth curve in the presence of synthetic peptides. Bacteria were grown in LB medium supplemented with 120 µM synthetic peptides. Growth was assessed in terms of OD_600_ measured at various time-points. Data represent mean values ± SE from three independent experiments. (**E**) Bacterial viability after 18 h of peptide treatment was determined by counting CFU in the cultures (500 µl). Data are the average of three independent experiments and mean values ± SE are represented.

The MgtR peptide was chemically synthesized (see Material and Methods) to investigate the effect of exogenous addition of peptide to wild-type *Salmonella*. MgtR-S17I peptide was also chemically synthesized, as an expected negative control, based on the results with endogenous production on intramacrophage replication (Fig. 1B). Both peptides, solubilized in Dimethyl sulfoxide (DMSO), were added at a final concentration of 120 µM to bacteria for 15 min before phagocytosis. Pretreatment with DMSO alone was used as control. Bacteria treated with MgtR peptide had a significantly lower intramacrophage survival than bacteria treated with DMSO (Fig. 3A). Surprisingly, bacteria treated with synthetic MgtR-S17I also exhibited a lower intramacrophage survival, to an extent even greater than the one observed with native MgtR (Fig. 3A), which noticeably contrasts with the result obtained with endogenous MgtR-S17I peptide (Fig. 1B).

We then examined the effect of the addition of synthetic peptides to *Salmonella* on MgtC protein level in liquid culture. Lysates were prepared from bacteria (Δ*mgtR* mutant) treated or untreated with peptides for 6 h. The level of MgtC protein decreased upon the addition of MgtR and the effect was even more dramatic in the presence of MgtR-S17I (Fig. 3B). The level of MgtC was normalized to that of the control cytoplasmic protein DnaK, which was similar in all conditions. Unexpectedly, the level of MgtB protein also reduced dramatically in the presence of both synthetic peptides (Fig. 3C), which again notably differs from the effect of endogenously produced peptides (Fig. 1D).

To address the mechanism of action of synthetic MgtR and MgtR-S17I peptides, we first investigated their effect on bacterial growth. Addition of synthetic peptides to bacterial cultures had no major effect on bacterial growth rate (Fig. 3D). Numeration of colony forming units (CFU) 18 h after addition of peptide confirmed the lack of effect on bacterial viability (Fig. 3E). Therefore, the effect of synthetic peptides on *Salmonella* intramacrophage survival is not due to a classical antimicrobial effect that would kill the bacteria or prevent their growth. Moreover, in the conditions used, peptides are not cytotoxic to J774 cells as shown by lactate dehydrogenase (LDH) release (Fig. S1).

Cumulatively, these results show a clear biological effect of the addition of synthetic peptides MgtR and MgtR-S17I exogenously to *S*. Typhimurium. Both synthetic peptides can lower *Salmonella* replication inside macrophages, without impairing bacterial growth in liquid culture. Despite the fact that addition of peptides reduced MgtC protein level dramatically, these peptides are likely to have other target(s) as suggested by reduction of MgtB protein. Thus, the findings obtained upon exogenous addition MgtR-derived peptides contrast with those obtained with endogenously expressed peptides, suggesting different mechanisms of action. Our findings also indicate that the Ala-coil motif is not required for the action of exogenous peptide, since this motif is disrupted in the MgtR-S17I peptide.

### Synthetic hydrophobic peptides act by lowering the level of multiple inner membrane proteins

Experiments carried out with *Salmonella* indicated a marked effect of addition of exogenous peptides on the level of MgtC and MgtB, which are both inner membrane proteins, but no effect on the cytoplasmic protein DnaK (Fig. 3). To decipher the mechanism of action of the synthetic peptides, we extended the analysis to other proteins, including outer membrane proteins as well as other inner membrane and cytoplasmic proteins. For this purpose, we took advantage of fusion of the T18 subunit of adenylate cyclase to the proteins of interest, which can be detected with anti-T18 antibodies. The fused proteins were expressed in *Escherichia coli*, an Enterobacteriaceae closely related to *S*. Typhimurium.

**Figure 4:**
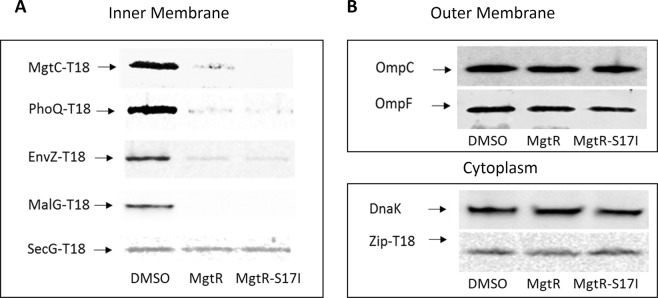
Synthetic peptides MgtR and MgtR-S17I decreased the level of various inner membrane proteins. (**A**) Effect of synthetic peptides on inner membrane proteins. The effect of synthetic peptides MgtR and MgtR-S17I added at 120 µM for 6 h on the stability of several inner membrane proteins (MgtC, PhoQ, EnvZ, MalG, SecG) fused to T18 was tested in *E*. *coli* BTH101 strain. Membranes were blotted with anti-T18 antibodies. (**B**) Effect of synthetic peptides on outer membrane and cytoplamic proteins. For analysis of outer membrane proteins (upper panel), western blot analysis was performed using anti-OmpC and anti-OmpF antibodies. In addition to DnaK, a cytoplasmic protein fused to T18 (zip-T18) was also tested using anti-T18 antibodies (lower panel). The experiment was repeated three times independently and one representative experiment is shown. Mouse anti-DnaK antibodies were used as loading control for all samples (not shown).

We first validated our approach by testing the effect of exogenous peptides on *E*. *coli* BTH101 strain expressing the recombinant *Salmonella* MgtC-T18 protein (Fig. 4A). Bacterial lysates were subjected to Western blot using anti-T18 antibodies. Upon addition of MgtR and MgtR-S17I peptides, the level of the MgtC-T18 recombinant protein in *E*. *coli* was reduced, exhibiting a pattern very similar to the one detected for MgtC protein in *Salmonella*. This result further suggests that the effect of peptides on MgtC and MgtC-T18 levels is not at the transcriptional level because in *E*. *coli*, MgtC-T18 was expressed from the *lac* promoter and not the *mgtC* promoter. We then tested the stability pattern of other unrelated inner membrane proteins fused to T18. PhoQ and EnvZ are both histidine kinases with two transmembrane helices, PhoQ being regulated by small membrane proteins MgrB and SafA^7,31^. MalG is a transporter with six transmembrane helices^32^ and SecG (three transmembrane helices) is part of the SecYEG translocon involved in translocation of proteins to the inner membrane^33^. The level of all proteins tested was lowered upon addition of both MgtR and MgtR-S17I, with the exception of SecG (Fig. 4A). On the other hand, the level of two outer membrane proteins, the OmpF and OmpC porins, remained unchanged upon addition of exogenous peptides (Fig. 4B). In addition, the level of a recombinant cytoplasmic protein (Zip-T18), classically used as control in BACTH experiments^34^, remained unaltered, which is consistent with the constant level observed for the cytoplasmic control protein DnaK.

Cumulatively, our results show that exogenous addition of MgtR-derived peptides decrease the level of several inner membrane proteins, whereas the level of cytoplasmic and outer-membrane proteins tested appeared unchanged.

### Implication of membrane-associated proteases in the biological activity of synthetic MgtR and MgtR-S17I peptides

**Figure 5:**
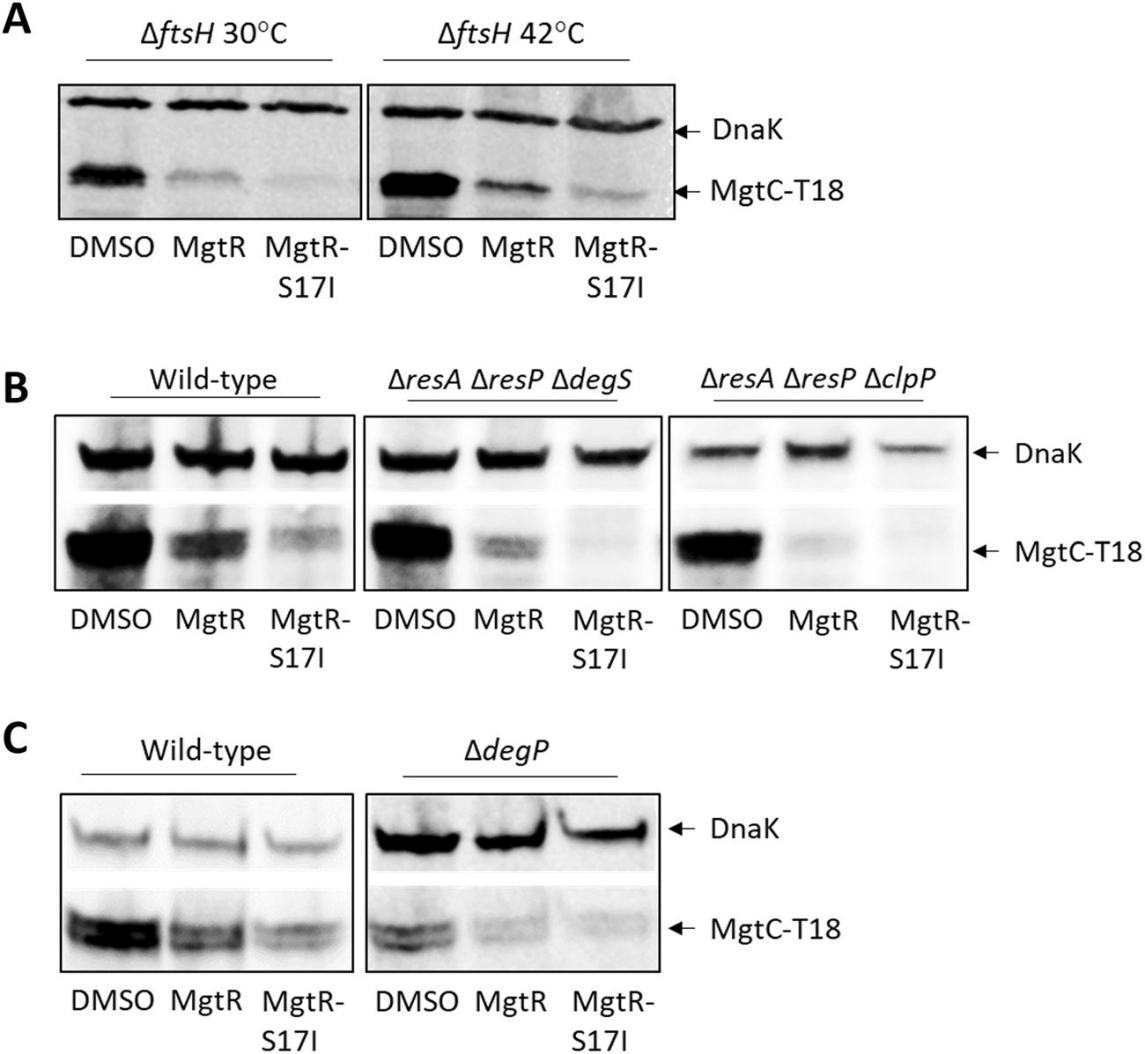
Implication of proteases in the effect of synthetic MgtR and MgtR-S17I peptides. *E*. *coli* mutants defective for diverse proteases were transformed with MgtC-T18 plasmid. Thermosentive *ftsH* mutant (**A**), triple mutants *rseA rseP degS* and *rseA rseP clpP* (**B**), as well as *degP* mutant (**C**) were used. The effect of synthetic peptides was compared between mutant strains and wild-type isogenic strains. Strains were cultured in LB medium supplemented with 120 µM of the synthetic peptides for 6 h at 37 °C except the thermosensitive *ftsH* mutant that was incubated at 30 °C or at the non-permissive temperature of 42 °C. Total extracts were blotted with anti-T18 antibodies and anti-DnaK antibodies as internal loading control.

Based on the current knowledge of regulatory membrane peptides, one can hypothesize that synthetic MgtR and MgtR-S17I peptides promote the degradation of multiple inner membrane proteins through a direct interaction between peptides and proteins, leading to unfolded target proteins, thus making them accessible for degradation. Alternatively, the synthetic peptides may somehow activate a protease involved in the stability of inner membrane proteins. Effect of exogenous peptides on protease activity within bacteria has been reported for acyldepsipeptide (ADEPs) antibiotics which deregulate proteolysis by ClpP^35,36^. Because endogenously expressed MgtR has been shown to promote MgtC degradation by FtsH, an AAA+ protease involved in the degradation of membrane proteins^6,37^, we first tested the implication of this protease in the biological effect driven by exogenous peptides. To determine the role of FtsH, we studied the level of MgtC-T18 protein in a thermosensitive *ftsH* mutant strain of *E*. *coli*, treated or non-treated with exogenous peptides, in comparison with the isogenic wild-type strain. At the non-permissive temperature of 42 °C, the level of MgtC-T18 expressed in the *ftsH* mutant, was found to be decreased upon treatment with MgtR or MgtR-S17I (Fig. 5A). Hence, a functional FtsH protease is not required to mediate the reduction of MgtC protein level in the presence of synthetic peptides.

We next examined the contribution of RseA, RseP and DegS, which are proteases with transmembrane domains involved in the regulation of membrane proteins^37^, as well as ClpP, using Δ*rseA* Δ*rseP* Δ*degS* and Δ*rseA* Δ*rseP* Δ*clpP* triple mutants of *E*. *coli*^38^. The profile of level of MgtC-T18 in the triple mutants, upon exogenous peptide addition, did not exhibit any rescue comparatively to the isogenic wild-type strain (Fig. 5B). We also considered the involvement of DegP protein, a multifunctional chaperone and a protease essential for clearance of denatured or aggregated proteins from the inner membrane and periplasmic space. The use of an *E*. *coli* Δ*degP* mutant, however, did not support the implication of DegP (Fig. 5C).

Our results clearly show a different biological effect of endogenous production of MgtR-S17I peptide from its exogenous addition. Unlike the endogenous peptide that is translocated from the cytoplasm to the inner membrane, the exogenous peptide may localize, at least transiently, in the periplasm. C-terminal peptides harboring YYF or YQF residues from misfolded outer membrane proteins located in the periplasm have been shown to activate DegS^39^. Notably, the C-terminal end of MgtR-S17I peptide is WQIVF. To address the contribution of this C-terminal end in the biological activity, we synthesized a shorter peptide that lacks the last five amino-acids (WQIVF) and tested its effect on the level of inner membrane protein MgtC-T18. This shorter peptide exhibited similar effect as the MgtR-S17I peptide (Fig. S2), indicating that the C-terminal end is dispensable for this biological effect. This result indicates that the mechanism of action of peptides does not mimic the process induced upon detection of misfolded outer membrane proteins in the periplasm, which is consistent with the finding that DegS is not implicated in the biological effect of the synthetic peptides (Fig. 5B).

To summarize, we could not correlate the decreased level of several inner membrane proteins upon bacterial treatment with MgtR and MgtR-S17I peptides with the activation of a specific protease among those tested. Although we cannot exclude the implication of another protease, an alternative hypothesis is that the peptides do not directly target protein degradation but rather target insertion of proteins in the inner membrane, possibly linked to an effect on the translocase system^40^. Interestingly, short cationic peptides rich in arginine and tryptophan have been reported to delocalize membrane proteins essential for respiration and cell-wall biosynthesis^41^. An effect on membrane protein level has however not been reported and their antimicrobial activity associated with bacterial cell death indicates that their mechanism of action differs from that of the peptides described in the present study.

### Amino-acid composition and structural features important for the action of synthetic peptides on inner membrane proteins

**Figure 6:**
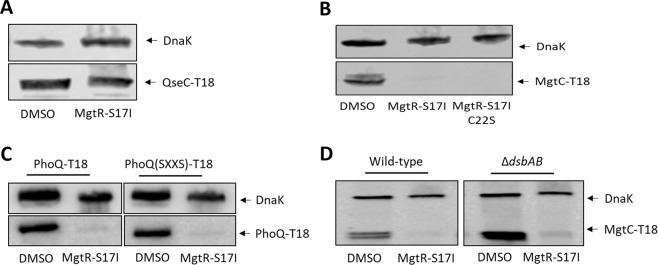
Role of cysteine residues in the biological activity of MgtR-S17I synthetic peptide. (**A**) Effect of MgtR-S17I on the stability of QseC-T18, QseC being a histidine kinase that lacks cysteine residues. (**B**) Biological activity of a synthetic peptide MgtR-S17I with a C22S residue (MgtR-S17I-C22S) on MgtC stability. (**C**) Effect of MgtR-S17I on mutated PhoQ-T18 that lacks cysteine residue (SXXS). (**D**) Effect of the DsbAB system on MgtR-S17I activity. The effect of synthetic peptide on the level of MgtC-T18 protein was compared between *dsbAdsbB* double mutant strain and wild-type isogenic strain. In all panels, membranes were blotted with anti-T18 antibodies and anti-DnaK antibodies as internal loading control.

By comparing the primary sequence of inner membrane proteins that are degraded by MgtR-S17I (MgtC, MgtB, PhoQ, EnvZ, MalG) and the one that resisted degradation (SecG), we noticed that the “resistant” SecG protein lacks cysteine residue, whereas “susceptible” proteins harbor at least one cysteine residue (not necessarily in the transmembrane domains). We tested the effect of MgtR-S17I peptide on QseC (also known as YgiY), a histidine kinase that has two transmembrane helices, like PhoQ and EnvZ, but lacks cysteine residue. Interestingly, QseC is not susceptible to degradation upon addition of MgtR-S17I peptide, as shown by monitoring the level of QseC-T18 recombinant protein (Fig. 6A). Taken together, these results suggested that the peptides act preferentially on inner membrane proteins containing cysteine residues.

A single cysteine residue is present in MgtR-S17I peptide (C22), which may play a role in the formation of disulfide bridges with target proteins. To test this hypothesis, we synthesized an MgtR-S17I peptide variant that harbors a serine residue at position 22 instead of cysteine (MgtR-S17I C22S). As shown in Fig. 6B, this peptide is as efficient as MgtR-S17I for degradation, indicating that the cysteine of the peptide does not contribute to its biological activity. We also tested the contribution of cysteine residues in proteins that are degraded upon addition of synthetic peptides. For this purpose, we replaced the two cysteine residues in PhoQ protein (CXXC motif) by serine residues (SXXS). The level of PhoQ-T18 lacking cysteine residues was similarly affected by MgtR-S17I peptide than the native protein (Fig. 6C), indicating that the presence of cysteine residues is not required to target PhoQ-T18 protein for degradation. Furthermore, we addressed the implication of the DsbAB system, which is involved in the introduction of disulfide bonds in cell envelope proteins^42,43^ by testing the activity of MgtR-S17I peptide in an *E*. *coli* strain carrying *dsbA* and *dsbB* mutations. Degradation of MgtC-T18 protein was retained in the Δ*dsbA* Δ*dsbB* double mutant (Fig. 6D), indicating that the DsbAB system is not involved in the biological activity of the synthetic peptide. Taken together, these results show that cysteine residues and disulfide bonds are not involved in the mechanism of action of MgtR-S17I peptide.

**Figure 7:**
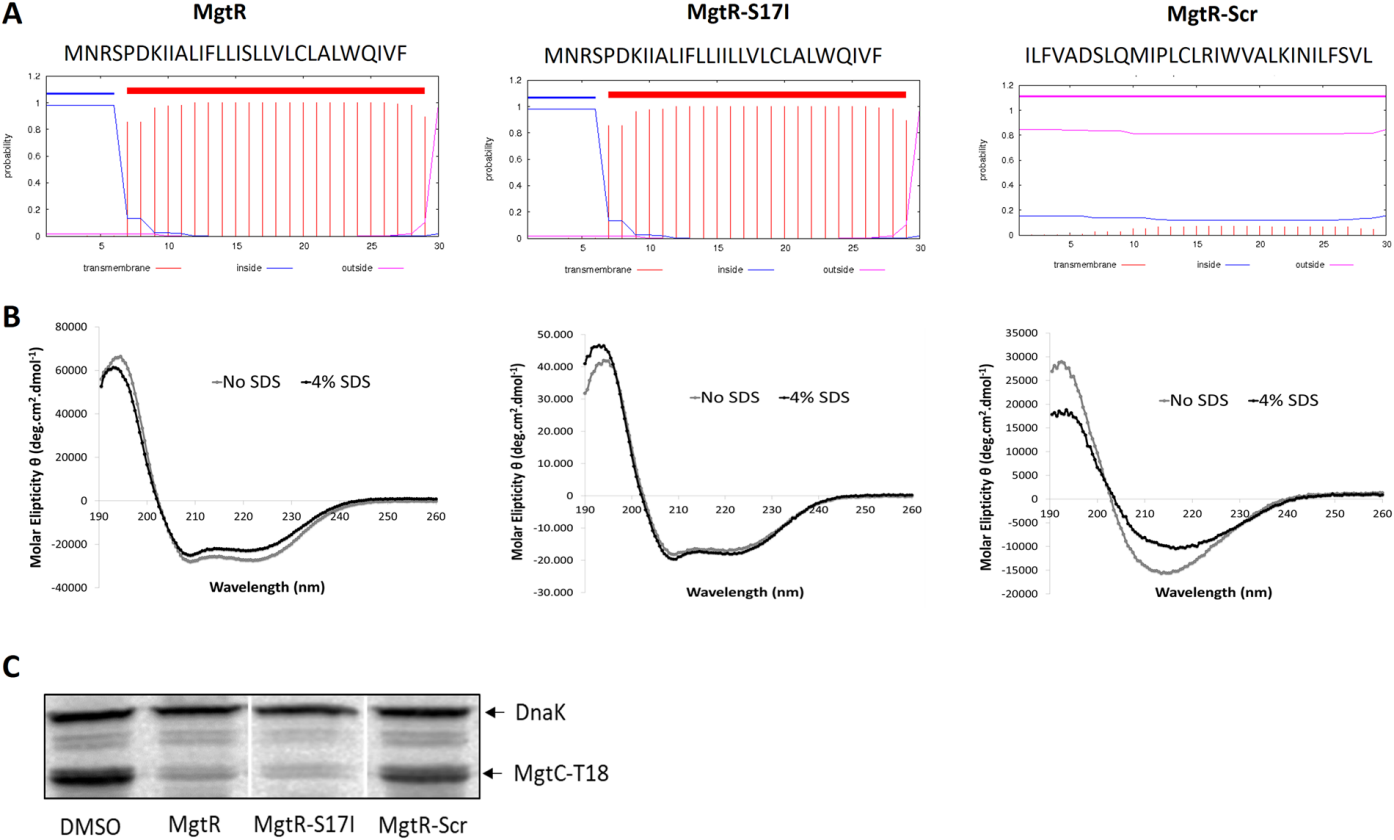
Role of the α-helix in the biological activity of MgtR peptide. (**A**) Sequence and transmembrane helix prediction for MgtR, MgtR-S17I and scrambled MgtR (Scr) peptides, using TMHMM program (http://www.cbs.dtu.dk/services/TMHMM/). (**B**) Circular dichroism spectra for MgtR, MgtR-S17I and Scr peptides in the absence of SDS (no SDS) or in the presence of 4% SDS. MgtR and MgtR-S17I display a spectra typical of α-helical structure, which is not the case for the Scr peptide. (**C**) Effect of scrambled MgtR peptide on MgtC-T18 level in comparison with MgtR and MgtR-S17I. Membrane was blotted with anti-T18 antibodies and anti-DnaK antibodies as internal loading control.

We next designed and synthesized a peptide that contained the amino-acids of MgtR in a scrambled order (Scr), thus retaining the hydrophobicity (73% hydrophobic residues), but lacking the predicted α-helix, as shown by the TMHMM program (Fig. 7A). As expected, the scrambled peptide exhibited a circular dichroism spectra that differs from that of MgtR and MgtR-S17I (Fig. 7B), indicative of a strong reduction of the α-helix structure. The α-helical content in the absence or presence of SDS was calculated with the CDPro Software package (CDSSTR, CONTINLL and SELCON3) as 93.7%/88.4%, 70.7%/70.8% and 37%/37% for MgtR, MgtR-S17I and scrambled MgtR, respectively (Fig. S3). Importantly, only a minor decrease of MgtC-T18 level was observed with this scrambled peptide used at 120 µM (Fig. 7C), suggesting that the biological effect of the synthetic MgtR peptide is linked to some extent to its α-helical structure, and not just to overall hydrophobicity.

### Concluding remarks

An increasing number of studies on membrane active peptides show that the peptides exert their biological activity by interacting with the cell membrane, either to disrupt it to lead to cell lysis (as antimicrobial peptides) or to translocate through it (as cell-penetrating peptides)^44^. In addition, external transmembrane domains mimicking native transmembrane domains can be used to specifically disrupt the folding of full length proteins or compete for intermolecular interactions^30^. Here we investigated the biological effect of synthetic hydrophobic peptides derived from MgtR on a bacterial pathogen and demonstrated an action that differs from that of endogenously produced peptides. While endogenous MgtR peptide targets MgtC and loses its activity upon disruption of helix-helix Ala-coil interacting motif (MgtR-S17I), exogenous MgtR and MgtR-S17I peptides act not solely on MgtC, but also lower the level of other inner membrane proteins, independently of the Ala-coil motif. The peptides may act specifically on inner membrane proteins in relation with their own localization in the inner membrane. The main characteristics of MgtR and MgtR-S17I peptides are indeed their hydrophobicity and linear helical structure^14^, and the α-helical structure appeared to be important to some extent for activity of the exogenous peptides. The MgtR-S17I peptide variant, which appears more efficient than MgtR peptide, may better insert within bacterial membrane. Importantly, these peptides act on membrane proteins without showing antibacterial activity, which contrasts with the action of classical antimicrobial peptides. Because several inner membrane proteins play a key role in virulence without being essential for bacterial growth, these synthetic hydrophobic peptides can display antivirulence properties. Interference with bacterial virulence is a promising alternative approach to traditional antimicrobial therapy that would apply less selective pressure to develop resistance and better preserve microbiota^15,16^. Further studies will be required to better characterize the mechanism of action of MgtR-related peptides and evaluate antivirulence potential of such molecules against various pathogens. We recently showed the biological activity of synthetic MgtR peptide against *P*. *aeruginosa*^45^, supporting the notion that the antivirulence effect can apply to diverse pathogens. Further work will also address the efficiency of such molecules in infected animal models and their potential immunomodulatory properties.

## Material and Methods

### Bacterial strains, plasmids and growth conditions

Bacterial strains used in this study are listed in Table 1. *S*. Typhimurium strains are derived from 14028s. *E*. *coli* thermosensitive *ftsH1* mutant strain was provided by T. Ogura^46^ and other *E*. *coli* protease mutant strains were provided by Y. Akiyama^38^. *E*. *coli* XL1Blue was used for cloning and *E*. *coli* BTH101 was used for BACTH experiments. Bacteria were grown in either Luria Broth (LB, Merck, France) supplemented with suitable antibiotics (Euromedex, France) such as ampicillin (Amp) at 100 μg.mL^−1^, kanamycin (Kan) at 50 μg.mL^−1^ and chloramphenicol (Cm) at 10 μg.mL^−1^ or NCE-minimal medium supplemented with 10 µM Mg^2+^ ^47^. Plasmids, listed in Table 2, were introduced in *S*. Typhimurium and *E*. *coli* strains by electroporation or chemical transformation^6^. Plasmids carrying the fusion proteins MgtC-T18, MalG-T18, T25-MgtR and T25-MgtRS17I have been described earlier^6,32^. For plasmids carrying MgtB-T18, PhoQ-T18, EnvZ-T18, SecG-T18 and QseC-T18, genes were amplified by colony PCR from *Salmonella* wild-type strain 14028 s with Red’Y’Gold Mix polymerase (Eurogentec) using primers listed in Table S1 and cloned into pUT18 vector. Site-directed mutagenesis was performed on plasmids using the Quickchange® II kit (Stratagene), according to manufacturer’s instructions.

**Table 1:** Strains used in this study.

Strain	Description	Reference or source
*S*. Typhimurium		
14028s	Wild type *S*. Typhimurium	EA Groisman
NM14	Δ*mgtC*	^47^
NM516	Δ*mgtR*	^6^
NM590	Δ*mgtR* pBBR1MCS	^6^
NM591	Δ*mgtR* pMgtR	^6^
NM1073	Δ*mgtR* pMgtRS17I	This study
NM667	14028s pBBR1MCS	^6^
NM668	14028s pMgtR	^6^
NM669	14028s pMgtRS17I	This study
*E*. *coli*		
BTH101	F- *cya-99 araD139 galE15 galK16 rpsL1* (Str^r^) *hsdR2 mcrA1 mcrB1*	^34^
AR3307	W3110 *zad220*::Tn10	^46^
AR3317	AR3307 *zgj3198*::Tn10 (kanR) *ftsH1*(Ts)	^46^
BW25113	Δ(araD-araB)567,ΔlacZ4787(::rrnB-3),λ-, rph-1, Δ(rhaD-rhaB)568, hsdR514	Keio collection
JW0157	BW25113 Δ*degP*775::kanR	Keio collection
AD16	Δ(pro-lac) thi/F’ lacIq ZM15 Y+ pro+	^51^
AD1840	AD16 Δ*resA*::cat Δ*resP*::Kan Δ*degS*::tet	^51^
KA306	AD16 Δ*resA* Δ*resP*::Kan Δ*clpP*::cat	^51^
JP114	McrA^−^ McrBC^−^ *Eco*K r^−^ m^−^ Mrr^−^	^52^
JP221	Δ*dsbB::Tet* Δ*dsbA::Kan (FRT)*	^52^

**Table 2:** Plasmids used in this study.

Description	Reference or source
pUT18 (Amp^R^)	^34^
pUT18-Zip	^34^
pUT18-MgtC	^6^
pUT18-MalG	^32^
pUT18-MgtB	This study
pUT18-PhoQ	This study
pUT18-PhoQ-SXXS	This study
pUT18-EnvZ	This study
pUT18-SecG	This study
pUT18-QseC (YgiY)	This study
pKT25 (Kan^R^)	^34^
pKT25-MgtR	^6^
pKT25-MgtRS17I	^6^
pBBR1MCS (Cm^R^)	^53^
pMgtR	^6^
pMgtRS17I	This study

### Peptide synthesis

Peptides were synthesized by a solid-phase method using the Fmoc methodology on an automated microwave peptide synthesizer (Liberty-1, CEM, Orsay, France) as previously described^29,45^. H-Rink amide ChemMatrix resin (100 micromoles, substitution 0.37 mmol.g^−1^, Longjumeau, France) was used to generate an amide function at the C-terminal end of all peptides. The MgtR peptide (MNRSPDKIIALIFLLISLLVLCLALWQIVF), MgtR-S17I single variant, MgtR-S17I-C22S double variant and MgtR-S17I lacking WQIVF at its C-terminal end (MgtR-S17I short) were synthesized following a double-coupling step for each amino acid (400 micromoles) activated with TBTU (500 micromoles). Additionally an acetylation step was applied at the end of each amino-acid incorporation to prevent the formation of incomplete peptides. After clivage from the resine and precipitation^45^, the final product was resuspended in an isopropanol/water (50%/50%: vol/vol) without any purification and analysed by MALDI-TOF mass spectrometry (Fig. S4). MgtR and MgtR-S17I peptides were synthesized two times independently and similar results were obtained from both synthesis. For experimentations, peptides were resuspended at a concentration of 3.2 mM in DMSO/water (50%/50%: vol/vol) as reported^45^. Using the same procedure, we also synthesized a scrambled peptide based on the amino-acid sequence of MgtR (permutation of the original peptide, ILFVADSLQMIPLCLRIWVALKINILFSVL)^45^.

For N-terminal labelling of the peptide with fluorescein, a spacer made with an aminohexanoic acid was first coupled to the N-terminal amino group as recommended^48^ to prevent the subsequent cyclization of the fluorescein with removal of the first amino acid. After coupling of the fluorescein, the peptide was processed as described above for the unlabeled peptide.

For C-terminal labelling of the peptide with fluorescein, a Fmoc-Lys(Aloc)-OH amino acid was first coupled on the resin as the C-terminal amino acid and the peptide was then synthesized as previously described. The Fmoc group was kept at the N-terminal end of the peptidyl resin until the labelling of the C-terminal end with fluorescein. At the end of the synthesis, the Aloc group was thus orthogonally removed by treating the resin following the tetrakis/phenylsilane procedure as previously described^49^. Once deprotected, the labelling of the epsilon-amine group of the C-terminal lysine was performed with a 10 fold excess of FITC and the resin was allowed for labelling overnight. Fmoc group was then removed and the peptide was dried prior final deprotection and cleavage step using standard conditions as described above.

### Circular dichroism (CD) spectra

CD spectra were recorded as described earlier^45^ on a Jasco 810 (Japan) dichrograph in quartz suprasil cells (Hellma) with an optical path of 1 mm using 40 µM peptide in solution in 30% isopropanol with 4% SDS (to mimic bacterial membranes) or without SDS. Spectra were obtained as before from 3 accumulations between 190 and 260 nm with a data pitch of 0.5 nm, a bandwidth of 1 nm and a standard sensitivity^45^. The crude spectra were then converted in molar ellipticity per residue and processed using CDPro software package and the programs CDSSTR, CONTINLL and SELCON3^50^.

### Association of FITC-MgtR with bacteria

For visualizing the association of FITC-MgtR peptide with bacteria, overnight culture of *S*. Typhimurium grown in LB was diluted 5 times in LB supplemented with 120 µM synthetic peptide and incubated at 37 °C for 1 h. After the incubation, bacteria were fixed with 4% paraformaldehyde in PBS for at least 1 h, washed three times with PBS and mounted on glass slides in Vectashield with 4’,6-diamidino-2-phenylindole (DAPI, from Vector Laboratories, Inc). The slides were examined using a Leica TCS SPE Confocal Laser Scanning Microscope (Leica, Leica-Microsystems, France) on the Montpellier Resources Imagerie (MRI) platform. All samples were scanned using a 63X/1.33 ACS Apo oil immersion objective lens. 405 and 488 nm lasers were used for excitation for DAPI and FITC respectively. DAPI was detected at 420–480 nm and FITC at 505–550 nm. The experiment was repeated three times.

### Macrophage infection

The murine macrophage cell line J774A.1 (ATCC TIB-67) was maintained at 37 °C in 5% CO_2_ in Dulbecco’s modified Eagle medium (DMEM) (Gibco) supplemented with 10% (v/v) fetal bovine serum (FBS) (Gibco). Macrophages were seeded at a density of 5 × 10^5^ cells per well in 24‐well plates and grown in DMEM supplemented with 10% (v/v) FBS and 5% CO_2_. Overnight cultures of bacterial strains, grown in LB, were washed in PBS and added to the macrophages at the multiplicity of infection (MOI) of 10. Macrophage infection was performed as described previously^47^. Replication rate was determined as the ratio of the number of bacteria at 18 h to that at 25 min post-phagocytosis.

To test the effect of synthetic peptides MgtR and MgtR-S17I on intramacrophage bacterial survival, *S*. Typhimurium strains were pre-treated with synthetic peptides (120 µM) in DMEM for 15 min at room temperature before infection.

### Viability assessment of bacteria treated with synthetic peptides

*S*. Typhimurium overnight culture grown in LB was diluted 5 times in LB supplemented with synthetic peptides (120 µM) or DMSO (blank) and incubated at 37 °C for 18 h. Bacterial growth was monitored by measuring the optical density at 600 nm (OD_600_) using a Tecan fluorimeter (SPARK 20 M) and bacterial viability was assessed after 18 h by CFU counting on LB agar plates.

### LDH assay

The cytotoxicity of synthetic peptides was assessed by release of LDH using the Pierce LDH cytotoxicity assay kit (Thermo Scientific). Macrophages were seeded in a 96 well plate and treated with peptides (120 µM) for 18 h. The assay was performed as described before^45^.

### Bacterial two-hybrid analysis

The BACTH system^34^ was used to evaluate the interaction between MgtC and MgtB proteins with MgtR and MgtR-S17I peptides. The plasmids used for the BACTH analysis are listed in Table 2. Recombinant plasmids carrying both T18 and T25 derivatives were introduced into BTH101. Transformed bacteria were grown at 30 °C in LB containing Amp at 100 μg.mL^−1^ and 1 mM Isopropyl β-D-1-thiogalactopyranoside (IPTG). The quantification of the interaction between the hybrid proteins was carried out with β-galactosidase assays as described previously^6^, where activity of β-galactosidase enzyme is expressed in arbitrary Miller units. Average values from at least three independent cultures performed in duplicates were plotted. A level of β-galactosidase activity at least five-fold higher than that of the control vectors is considered as indicative of an interaction between the protein partners^34^.

### Preparation of lysates from bacterial cultures treated with synthetic peptide

Overnight culture of *S*. Typhimurium grown in NCE medium containing 10 μM MgCl_2_^19^ was diluted 5 times in the same medium supplemented with synthetic peptides (120 µM) and incubated for 6 h at 37 °C before harvesting. Overnight cultures of *E*. *coli* strains carrying T18 fusion proteins, grown in LB Broth containing 100 μg.mL^−1^ ampicillin, 0.5 mM IPTG, were diluted 5 times in the same medium supplemented with synthetic peptides (120 µM) and incubated for 6 h at 30 °C (or 37 °C as indicated in the text) before harvesting. For *E*. *coli* thermosensitive *ftsH1* mutant transformed with MgtC-T18 plasmid, bacteria were first incubated for 30 min at 42 °C (non-permissive temperature) and then incubated with the synthetic peptides for 6 h at 42 °C. To prepare whole-cell extracts, cultures were normalized to OD_600_, centrifuged, resuspended in Laemmli buffer and lysed by heating at 95 °C for 5 min before performing Western Blot assay.

### Western blot analysis

Samples were electrophoresed on 4–12% pre-cast ExpressPlus^TM^ PAGE Gels (Genscript) and transferred onto nitrocellulose membrane (Invitrogen) for immunoblotting. Rabbit anti-MgtC^6^ and rabbit anti-MgtB antibodies were used at 1:500 and 1:1000 dilution respectively. Mouse anti-DnaK antibody (Tebubio) was used at 1:5000 dilution, whereas mouse anti-T18 antibody (monoclonal antibody sc-13582, Santa-Cruz) was used at 1:1000 dilution. Rabbit anti-OmpF and anti-OmpC antibodies were used at 1:1000. Anti-rabbit antibody conjugated to horseradish peroxidase (Sigma) was used at 1:5000 dilution for the detection of MgtC, using Western Lightning® Plus-ECL (PerkinElmer Millipore). Anti-mouse or anti-rabbit antibody labeled with fluorescent dye DyLight 800 (Thermoscientific) were used for the detection of T18, DnaK, MgtB, OmpF and OmpC using LICOR Odyssey Fc Imaging System (excitation 783 nm and emission 797 nm) to quantify the amount of protein levels.

### Statistical analysis

Statistical analyses were performed by using the unpaired Student’s t-test. Only P-values < 0.05 were considered as statistically significant.

## Supplementary information

Supplementary figures S1, S2, S3, S4

Supplementary information for Western blots (uncropped blots)

Supplementary information for MALDI-TOF (full spectra)
