# Supplementary material for: Synthetic hydrophobic peptides derived from MgtR weaken Salmonella pathogenicity and work with a different mode of action than endogenously produced peptides
Source: Sci Rep. 2019 Oct 24;9:15253. doi: 10.1038/s41598-019-51760-2 (PMC6813294; doi:10.1038/s41598-019-51760-2)
Supplement: Supplementary file 1 — Supplementary figures S1, S2, S3, S4 [file 41598_2019_51760_MOESM1_ESM.pdf]

Synthetic hydrophobic peptides derived from MgtR weaken *Salmonella* pathogenicity and work with a different mode of action than endogenously produced peptides

Mariana Rosas Olvera, Preeti Garai, Grégoire Mongin, Eric Vivès, Laila Gannoun-Zaki, Anne-Béatrice Blanc-Potard

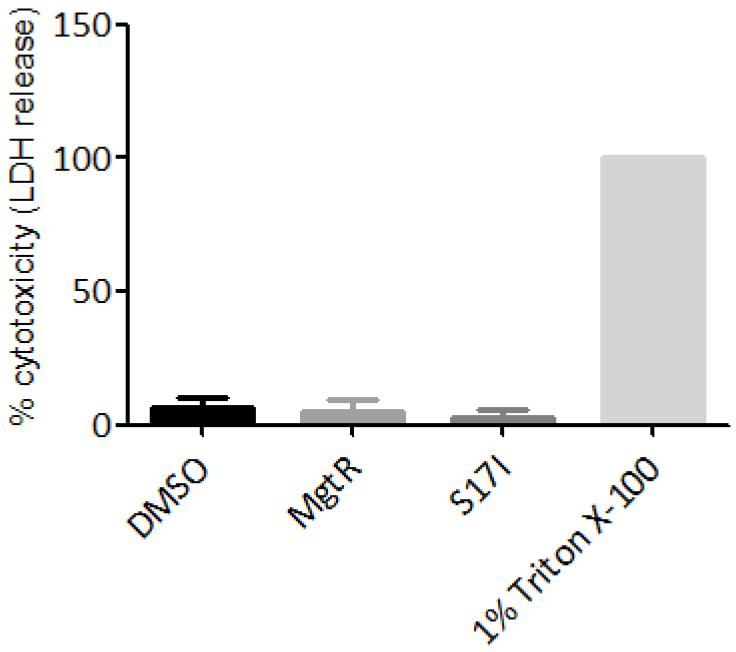

Fig. S1. Assessment of cytotoxicity of peptides toward macrophages using LDH release assay. J774 cells were treated with 120 µM peptides or 50% DMSO (solvent control) in DMEM, for 18 hours at 37°C and 5% CO_2. LDH released by the macrophages in the medium was quantified , normalized to DMEM medium control and plotted as percent cytotoxicity or LDH release, where the amount of LDH released by cells lysed by 1% Triton X-100 was considered as 100%.

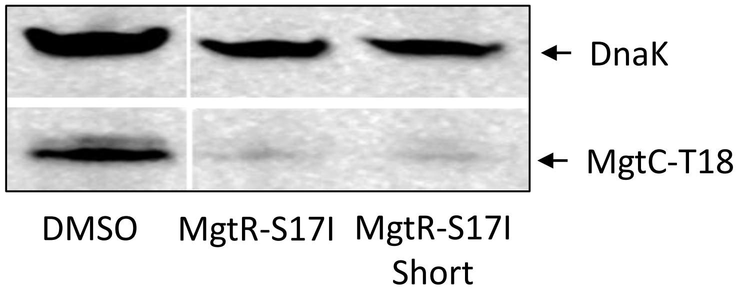

Fig. S2. Role of aromatic residues at the C-terminus in the biological activity of MgtR-S17I peptide. Biological activity of a synthetic peptide lacking the aromatic residues at the C-terminus (MgtR-S17I Short) on MgtC stability. Membrane was blotted with anti-T18 antibodies and anti-DnaK antibodies.

MgtR

Used Program (CD Pro Package)	Alpha-Helical content Without/With 4% SDS	Beta-Sheet content Without/With 4% SDS	Unordered content Without/With 4% SDS
**CDSSTR**	88.7/90.6	0/1.5	11.3/7.9
**CONTINLL**	91.8/82.1	1.5/0.8	5.8/17
**SELCON3**	100.6/92.6	-6.8/5.2	12.6/14.4

MgtR-S17I

Used Program (CD Pro Package)	Alpha-Helical content Without/With 4% SDS	Beta-Sheet content Without/With 4% SDS	Unordered content Without/With 4% SDS
**CDSSTR**	79.6/77.6	5.9/5.5	13.5/15.5
**CONTINLL**	66.7/67.6	2.7/3.6	30.5/28.9
**SELCON3**	65.8/67.2	3.6/3.8	29.3/29.3

MgtR-Scr

Used Program (CD Pro Package)	Alpha-Helical content Without/With 4% SDS	Beta-Sheet content Without/With 4% SDS	Unordered content Without/With 4% SDS
**CDSSTR**	40.8/40.9	23/20.1	36/38.4
**CONTINLL**	34/34	21.3/21.3	44.8/44.8
**SELCON3**	36.1/36.1	16/16	49.5/49.5

Fig. S3. Percentage of alpha-helix in the peptides. The crude circular dichroïsm spectra without or with SDS were converted in molar ellipticity per residue and processed using CDPro software package programs CDSSTR, CONTINLL and SELCON3. Content in Alpha-helical, Beta-Sheet and Unordered structures is expressed in percentage without or with SDS.

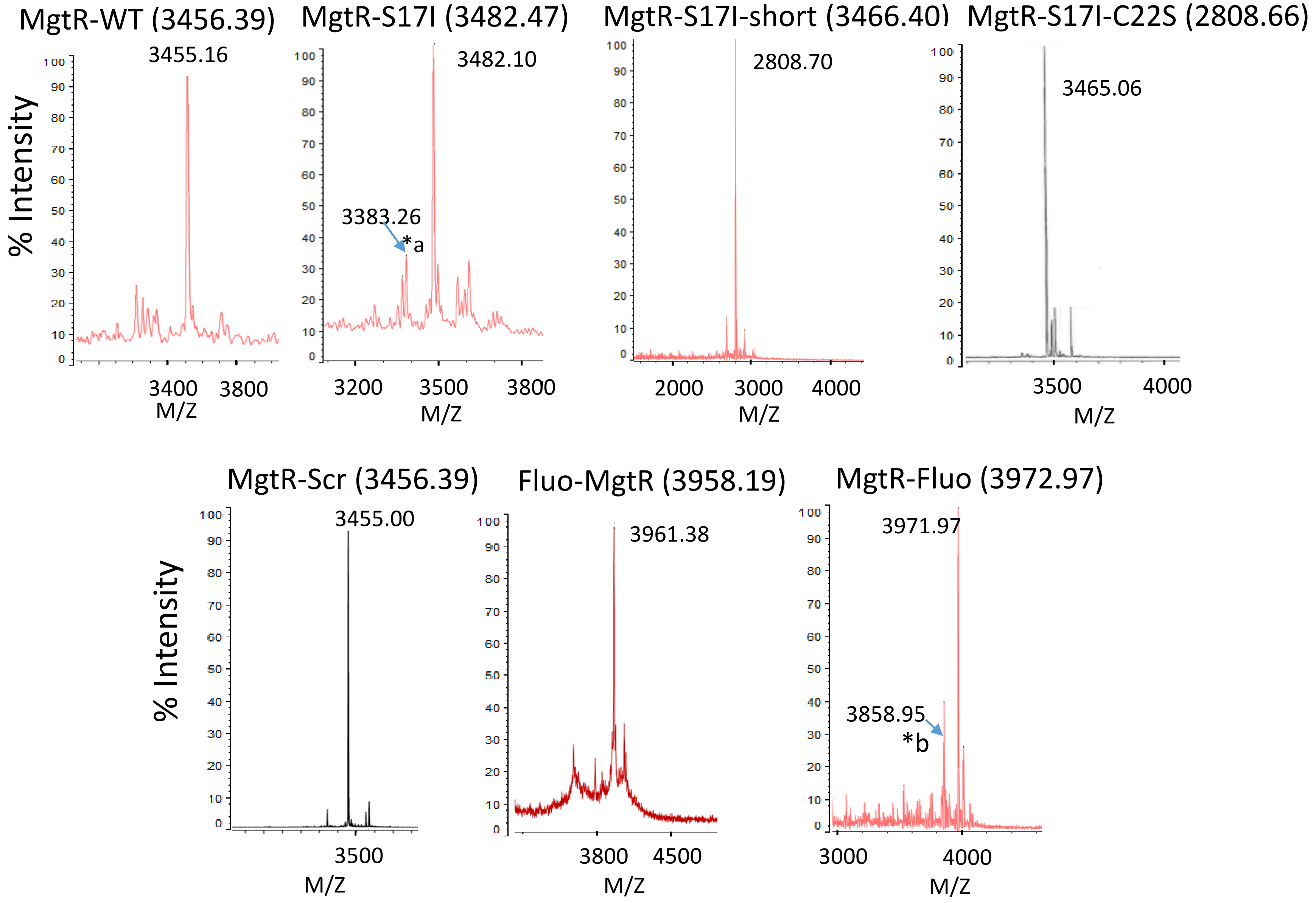

Fig. S4. Mass spectrometry analysis of the MgtR-derived peptides by MALDI-TOF. In all cases, the major peak corresponds to the expected mass (indicated in brackets) and represent more than 85% of the signal intensity. For some peptides, a minor contamination could be detected: a deletion of one single valine residue in the peptide MgtR-S17I (*a :delta mass of 99 Da) and to a deletion of one single leucine/isoleucine in the MgtR-Fluo peptide (*b :delta mass of 113 Da).
