# Supplementary material for: Synthetic hydrophobic peptides derived from MgtR weaken Salmonella pathogenicity and work with a different mode of action than endogenously produced peptides
Source: Sci Rep. 2019 Oct 24;9:15253. doi: 10.1038/s41598-019-51760-2 (PMC6813294; doi:10.1038/s41598-019-51760-2)
Supplement: Supplementary file 2 — Supplementary information for Western blots (uncropped blots) [file 41598_2019_51760_MOESM2_ESM.pdf]

Synthetic hydrophobic peptides derived from MgtR weaken *Salmonella* pathogenicity and work with a different mode of action than endogenously produced peptides

Mariana Rosas Olvera, Preeti Garai, Grégoire Mongin, Eric Vivès, Laila Gannoun-Zaki, Anne-Béatrice Blanc-Potard

Figure 1C

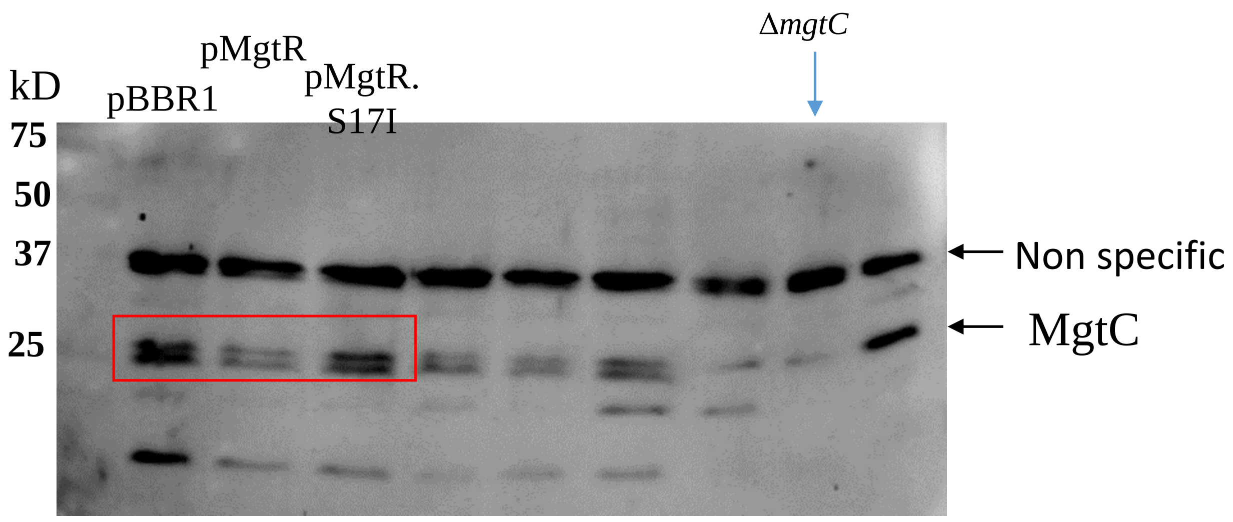

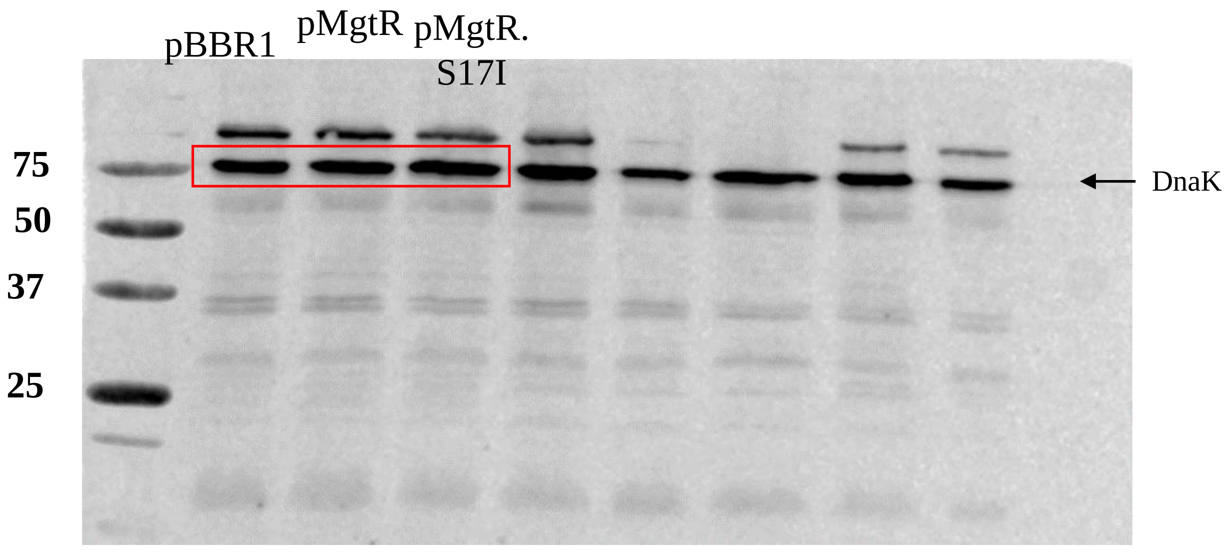

Gel 30/03/17
SDS PAGE 12,5%
Ac anti-MgtC 1/500 (anti-rabbit)

Figures 1D and 3C

14/02/18
SDS PAGE 12,5%

anti MgtB 1/1000

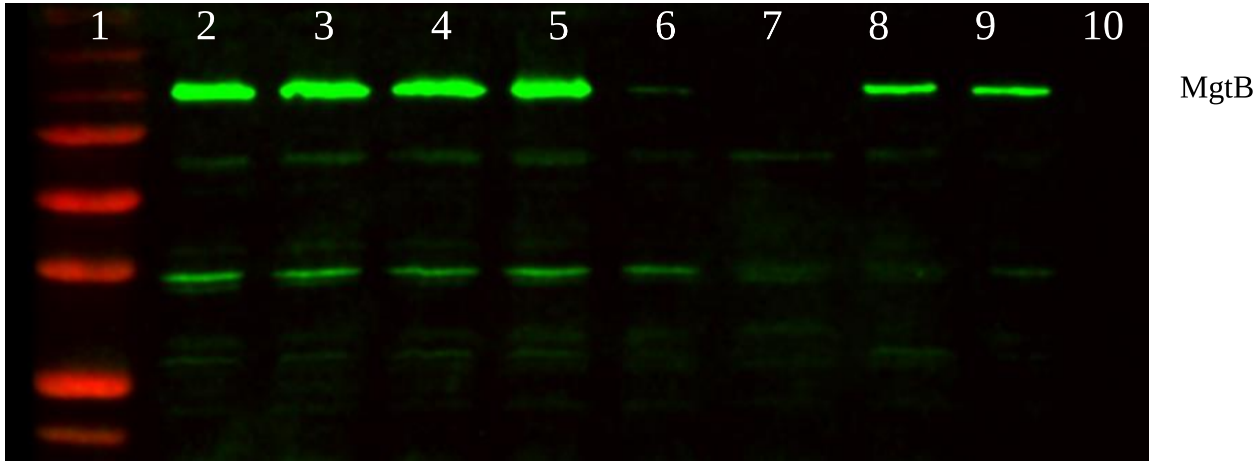

1 M
2 Δ *mgtR*: pBBRR
3 Δ *mgtR*: pMgtR
4 Δ *mgtR*: pMgtR.S17I
5 Δ *mgtR*: DMSO
6 Δ *mgtR*: MgtR
8 Δ *mgtC*

anti DnaK 1/5000

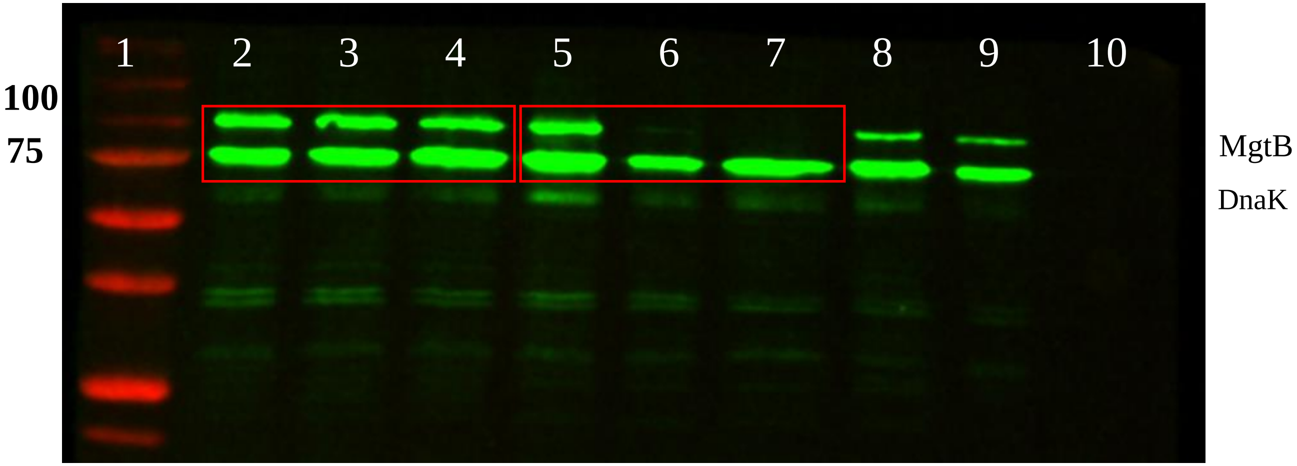

Figure 3B

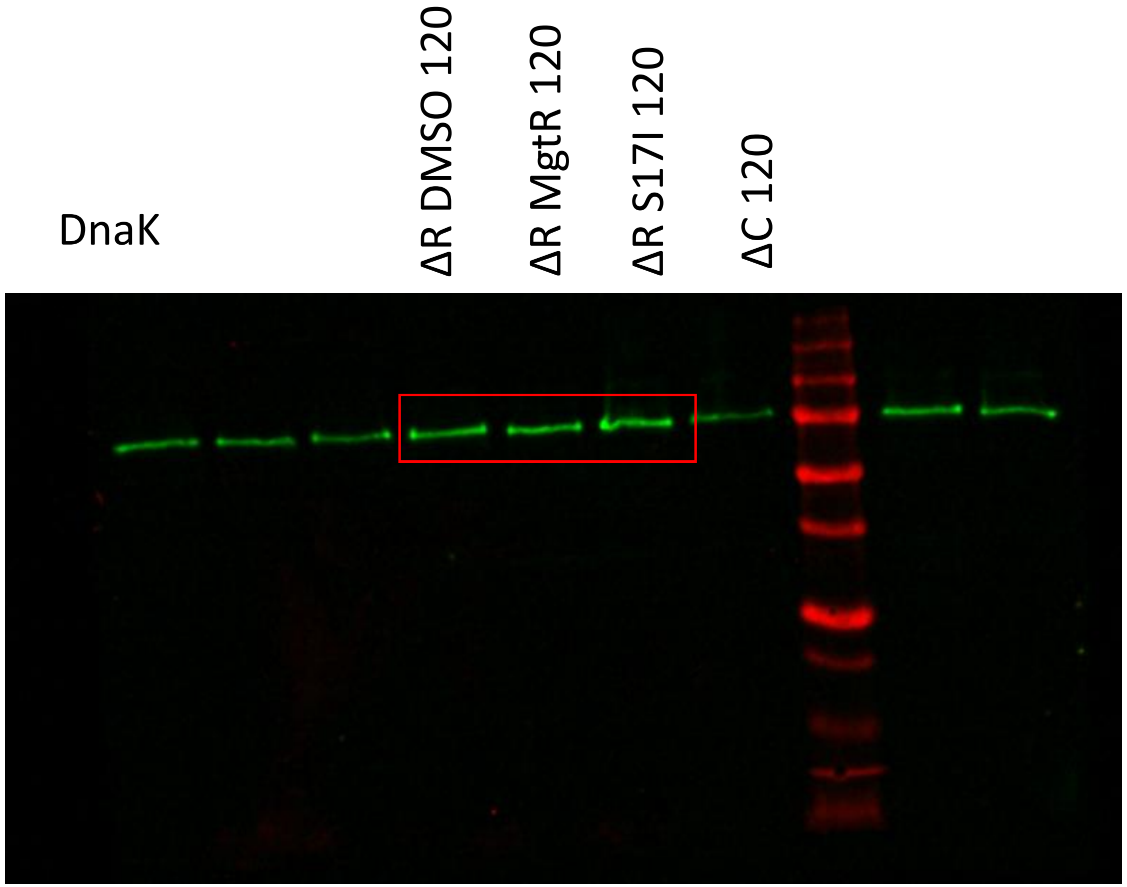

Western 03 et 08/02/17
SDS PAGE 12,5%
Ac HRP 1:5000 (rabbit) 1st use

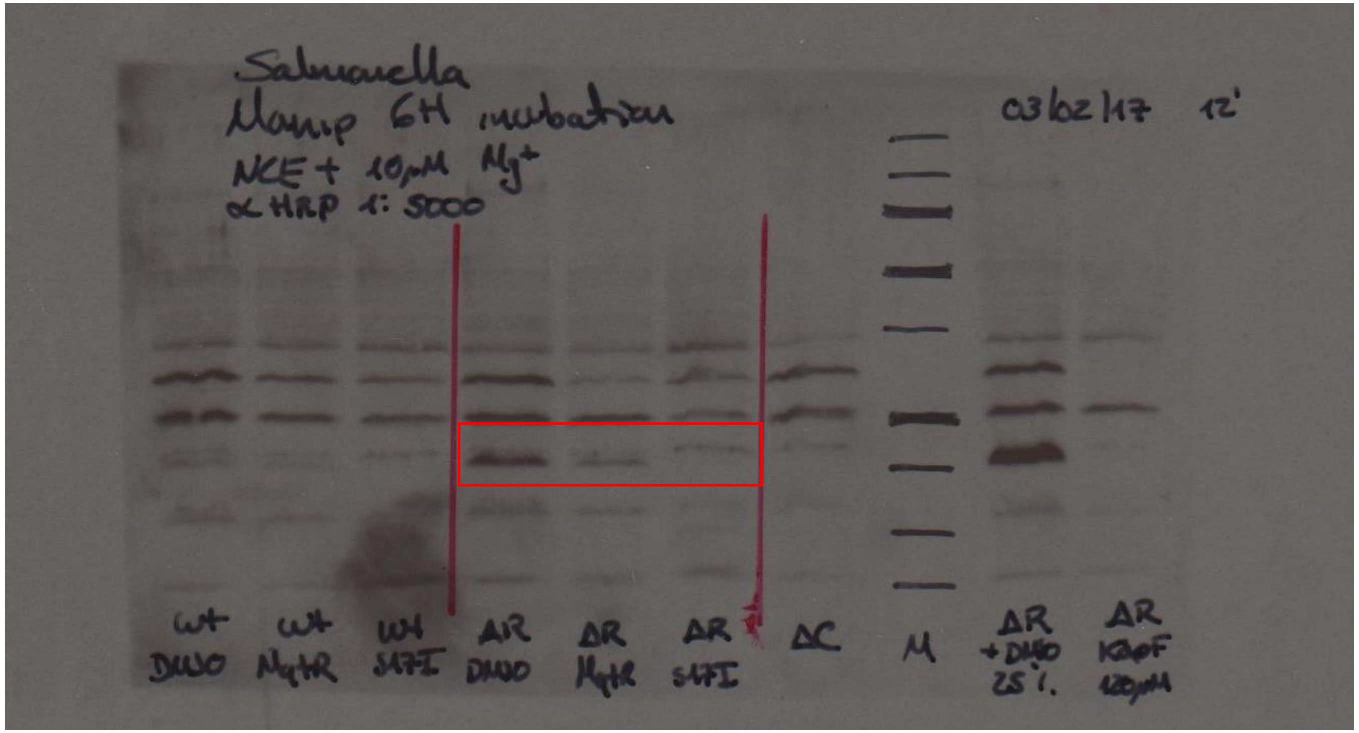

Figure 4 A
MgtC-T18
MalG-T18

06/07/16
SDS PAGE 12,5%
Ac anti-T18 1/2000 +anti DnaK 1/5000 (anti-mouse)

1 2 3 4 5 6 7 8 9 10

kD
75
50
37
25

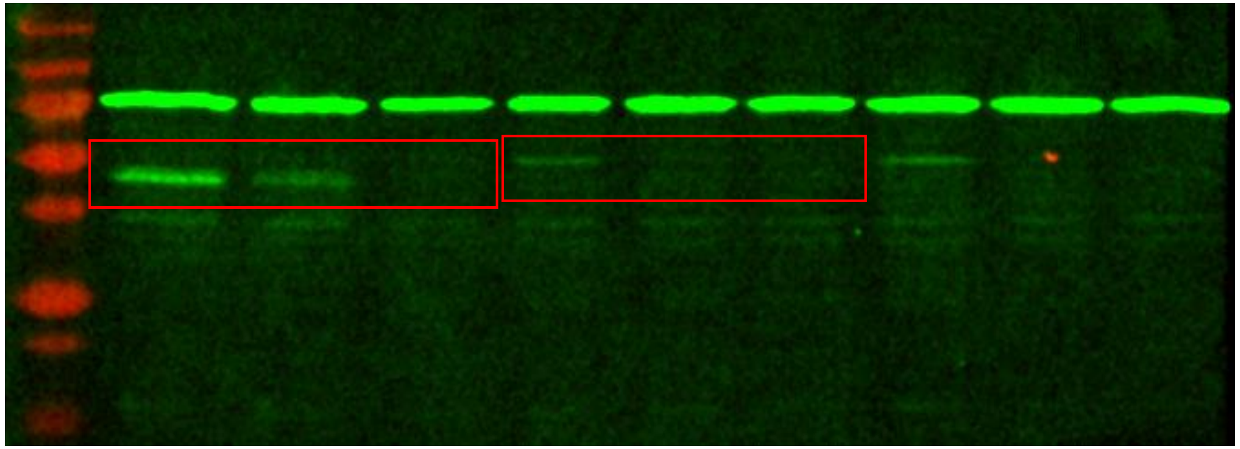

1 Marker
2 MgtC St-18 + DMSO
3 MgtC St-18 + MgtR
4 MgtC St-18 + S17I
5 MalG St-18 + DMSO
6 MalG St-18 + MgtR
7 MalG St-18 + S17I

MgtC 25kD +T18= 43kD
MalG 296aa 32kD +T18= 50kD

Figure 4 A
EnvZ-T18

29/06/16
SDS PAGE 12,5%
Ac anti-T18 1/2000 (anti-mouse)

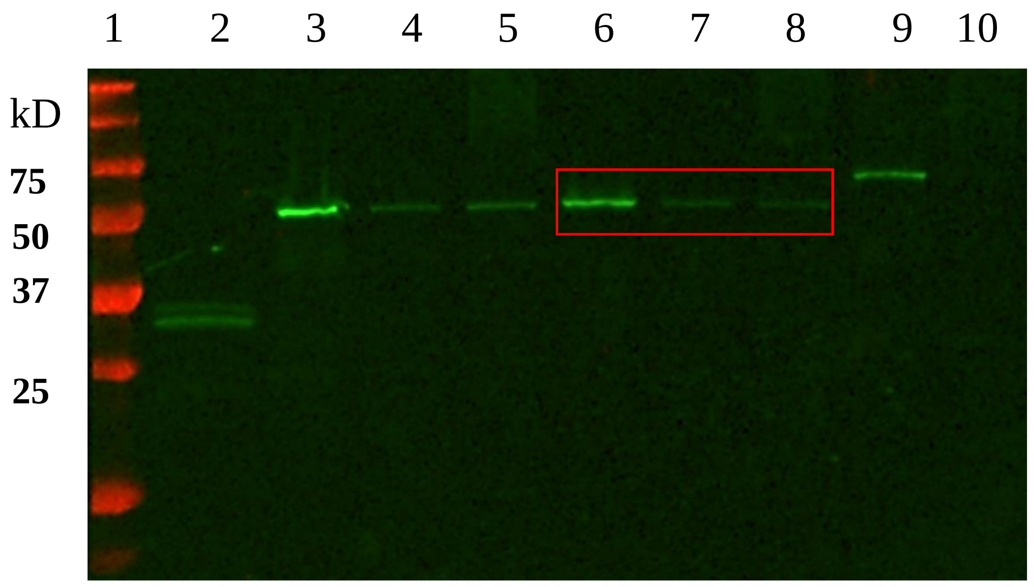

1 Marker

6 EnvZ St-18 + DMSO
7 EnvZ St-18 + MgtR
8 EnvZ St-18 + S17I

EnvZ 450aa 50kD +T18= 68kD

Figure 4 A
PhoQ-T18

10/05/17
SDS PAGE 12,5%
Ac anti-T18 1/2000 - anti DnaK 1/5000

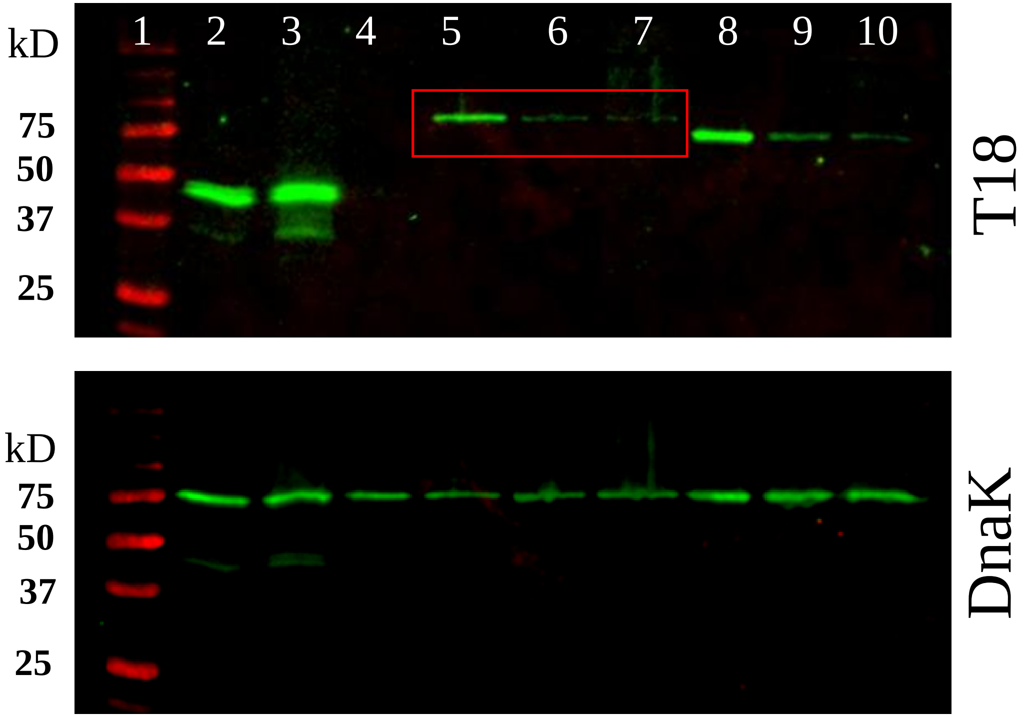

5 PhoQ St-18 + DMSO
6 PhoQ St-18 + MgtR
7 PhoQ St-18 + S17I

PhoQ 487aa 53kD +T18= 71kD

Figure 4 A
SecG-T18

3/07/17
SDS PAGE 12,5%
Ac anti-T18 1/2000 - anti DnaK 1/5000 new Preeti

kD

75

50

37

25

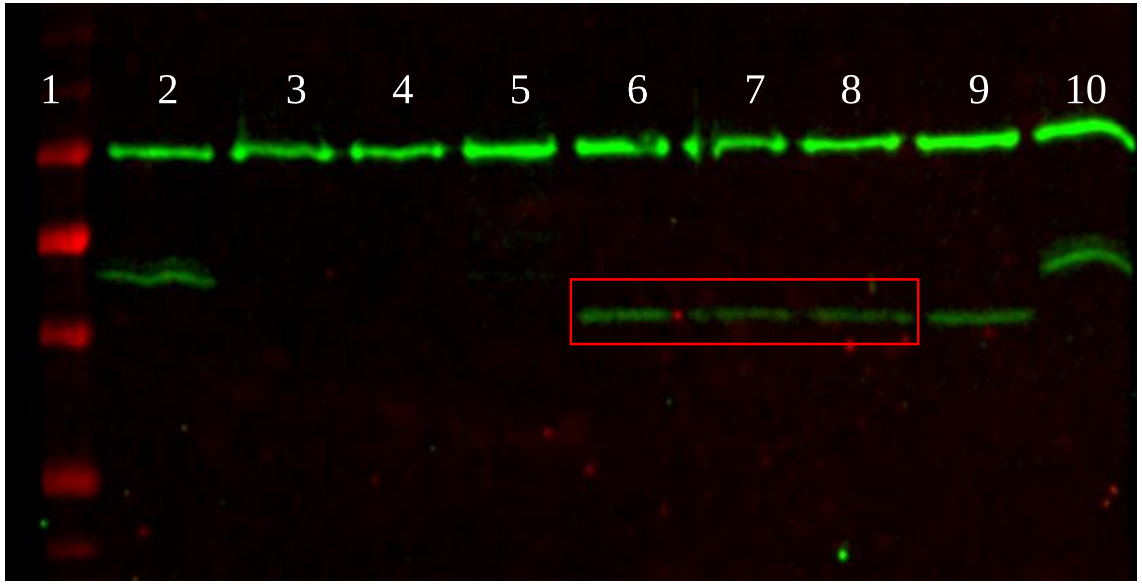

1 **M**

6 SecG St T18 + DMSO
7 SecG St T18 + MgtR
8 SecG St T18 + S17I

Figure 4B OmpC

16/02/18
SDS PAGE 12,5%
BTH101:MgtC-T18

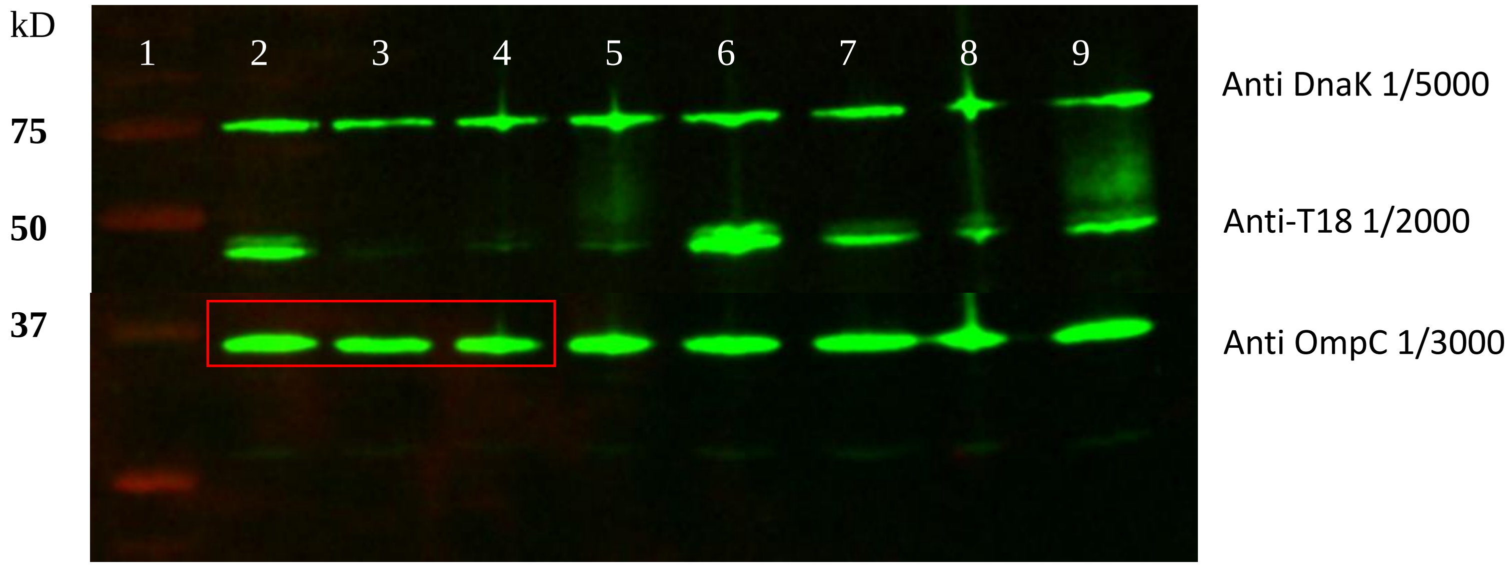

1 M
2 BTH101: MgtC St-18 DMSO
3 BTH101: MgtC St-18 MgtR
4 BTH101: MgtC St-18 S17I

Figure 4B OmpF

Gel 30/03/17
SDS PAGE 12,5%
Ac anti-OmpF 1/1000 (anti-rabbit)

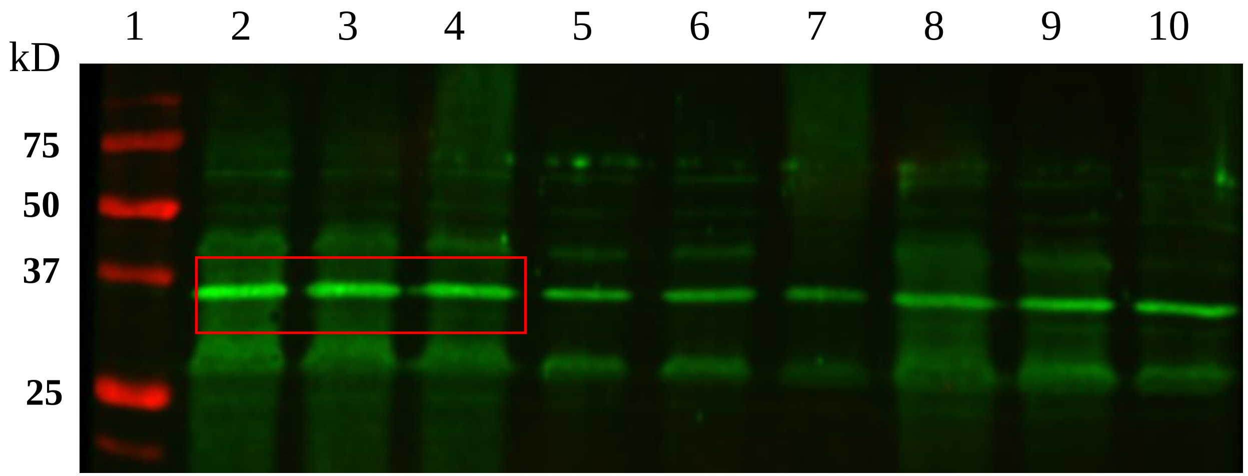

1 Marker
2 MgtC St-18 + DMSO
3 MgtC St-18 + MgtR
4 MgtC St-18 + S17I

OmpF 363aa 35kD

Figure 4B
Zip-T18

21/07/17
SDS PAGE 12,5%
Ac anti-T18 1/2000 - anti DnaK 1/5000

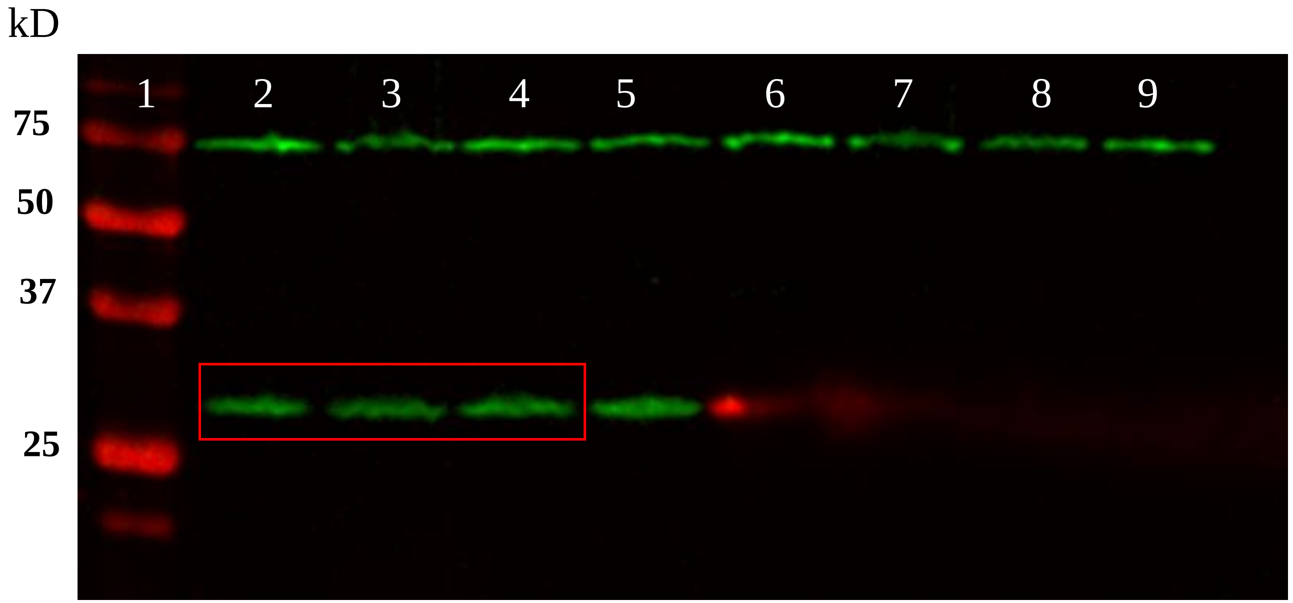

1 **M**
2 Zip St T18 + DMSO
3 Zip St T18 + MgtR
4 Zip St T18 + S17I

Figure 4B
DnaK / MalG

06/04/18
SDS PAGE Pre-cast 4-12%
BTH101 samples 03-04-18

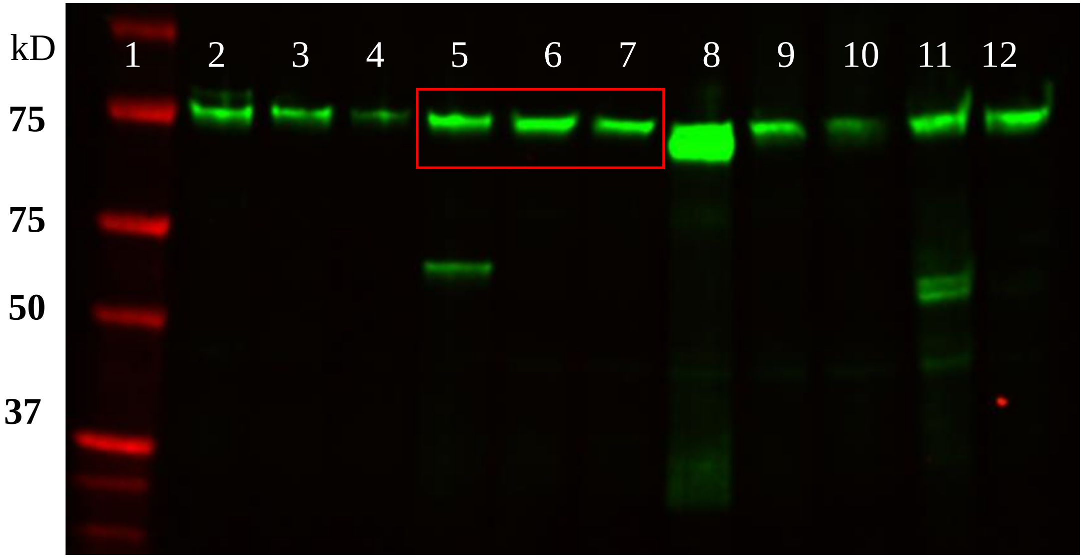

Anti-DnaK 1/5000

1 M
5 BTH101: MalG St-18 DMSO
6 BTH101: MalG St-18 MgtR
7 BTH101: MalG St-18 S17I

Fig5A

Folder 0012435_01
SDS PAGE 12,5%
Ac anti-T18 1/2000 - anti DnaK 1/5000

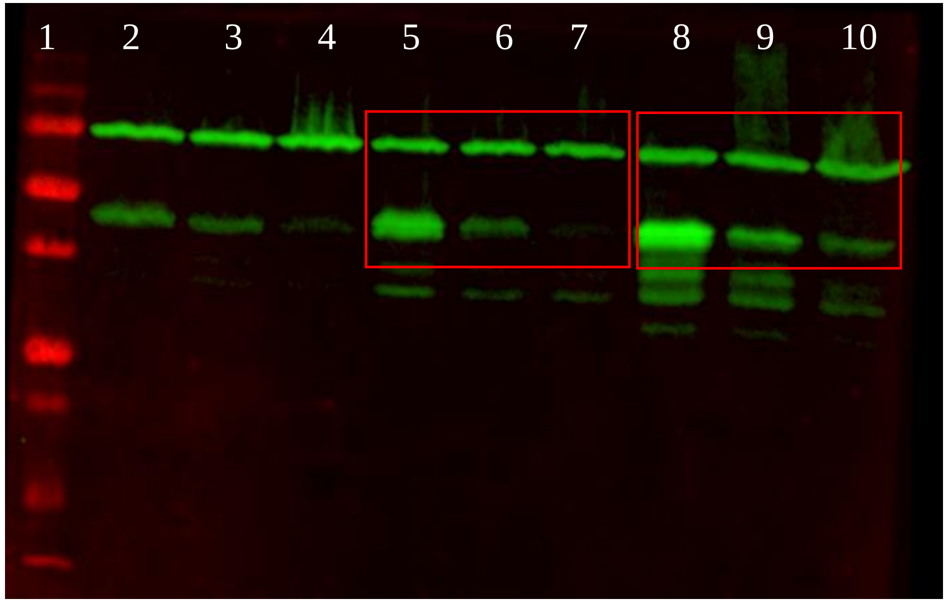

1 Marker
5 mut ftsH 30C MgtC St-18 + DMSO
6 mut ftsH 30C MgtC St-18 + MgtR
7 mut ftsH 30C MgtC St-18 + S17I
8 mut ftsH 42C MgtC St-18 + DMSO
9 mut ftsH 42C MgtC St-18 + MgtR
10 mut ftsH 42C MgtC St-18 + S17I

Figure 5B

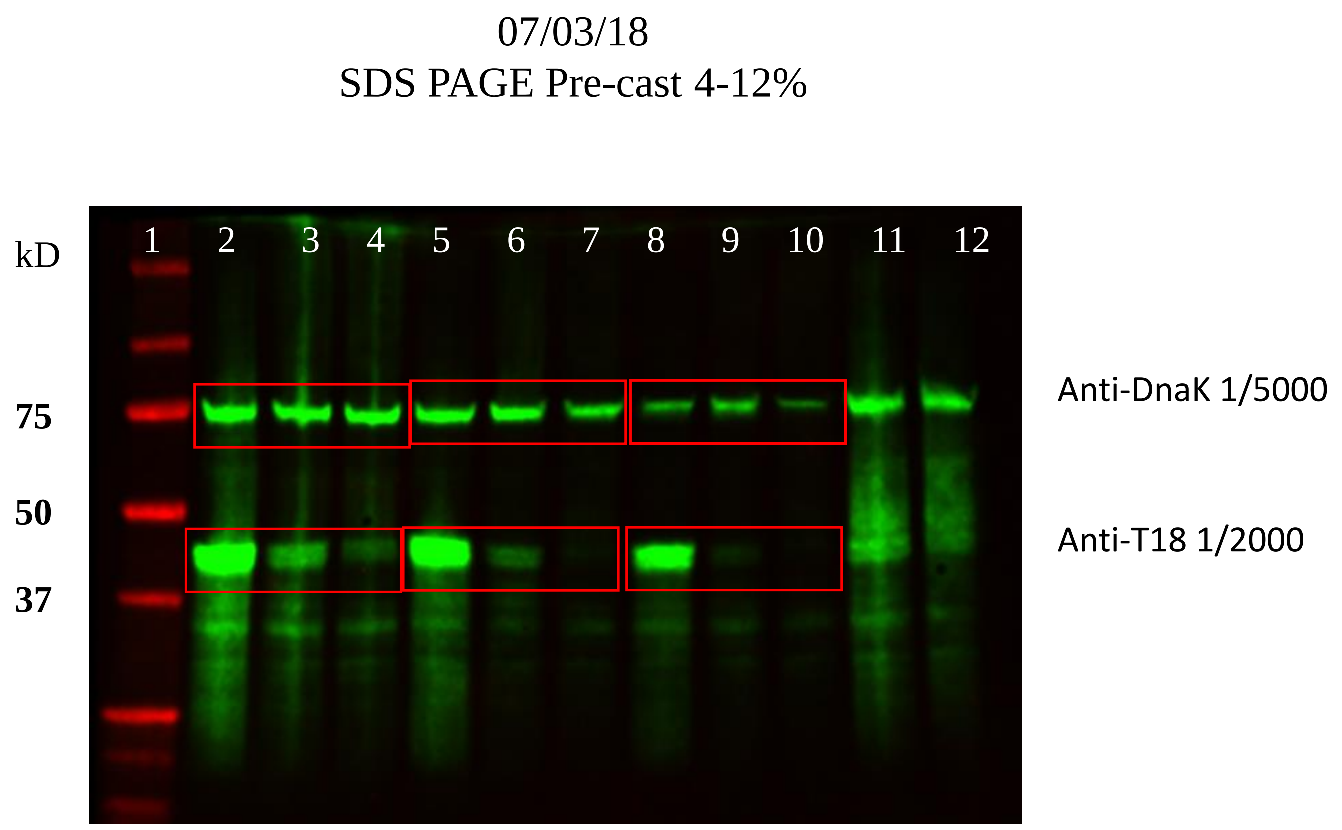

1 M
2 AD16: MgtC St-18 DMSO
3 AD16: MgtC St-18 MgtR
4 AD16: MgtC St-18 S17I

5 AD1840: MgtC St-18 DMSO
6 AD1840: MgtC St-18 MgtR
7 AD1840: MgtC St-18 S17I

8 KA306: MgtC St-18 DMSO
9 KA306: MgtC St-18 MgtR
10 KA306: MgtC St-18 S17I

AD16= wild-type
AD1840=Δ*resA* Δ*resP* Δ*degS*
KA306=Δ*resA* Δ*resP* Δ*clpP*

Figure 5C

06/04/18
SDS PAGE Pre-cast 4-12%
30°C 6h culture Wt et ΔdegP samples 05-04-18

Anti-DnaK 1/5000

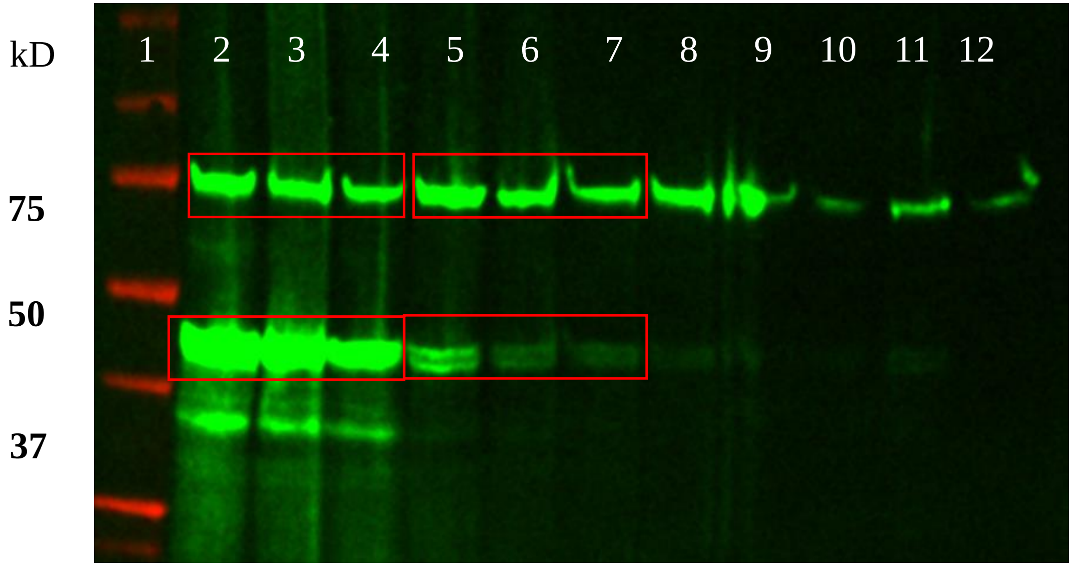

Anti-T18 1/2000

1 M
2 Wt Keio: MgtC-T8 DMSO
3 Wt Keio: MgtC-T8 MgtR
4 Wt Keio: MgtC-T8 S17I
5 ΔdegP: MgtC St-18 DMSO
6 ΔdegP: MgtC St-18 MgtR
7 ΔdegP: MgtC St-18 S17I

Figure 6A

Western Blot anti T18+DnaK (QseC-T18)

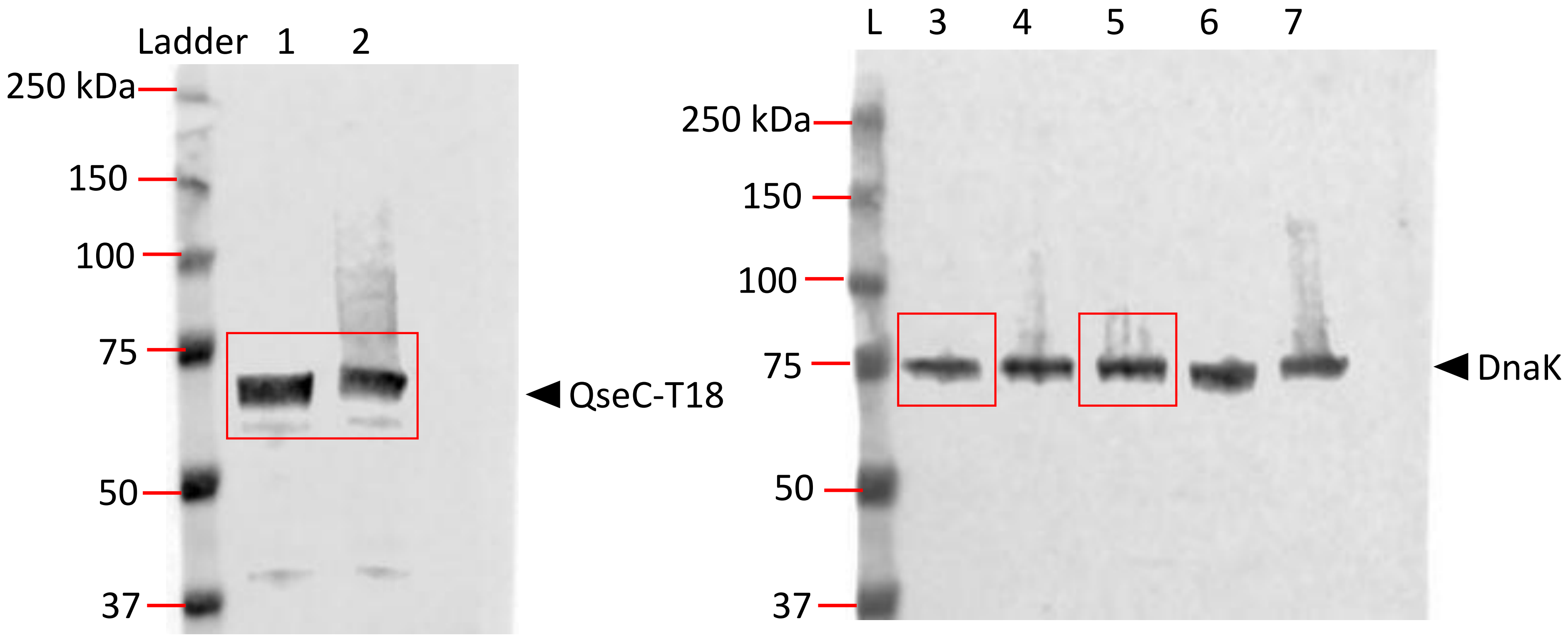

Ladder
1 BTH101 pQseC-T18 + DMSO
2 BTH101 pQseC-T18 + MgtRS17I
05/07/2018

Ladder
3 BTH101 pQseC-T18 + DMSO
4 BTH101 pQseC-T18 + MgtR
5 BTH101 pQseC-T18 + MgtRS17I
05/07/2018

Figure 6B

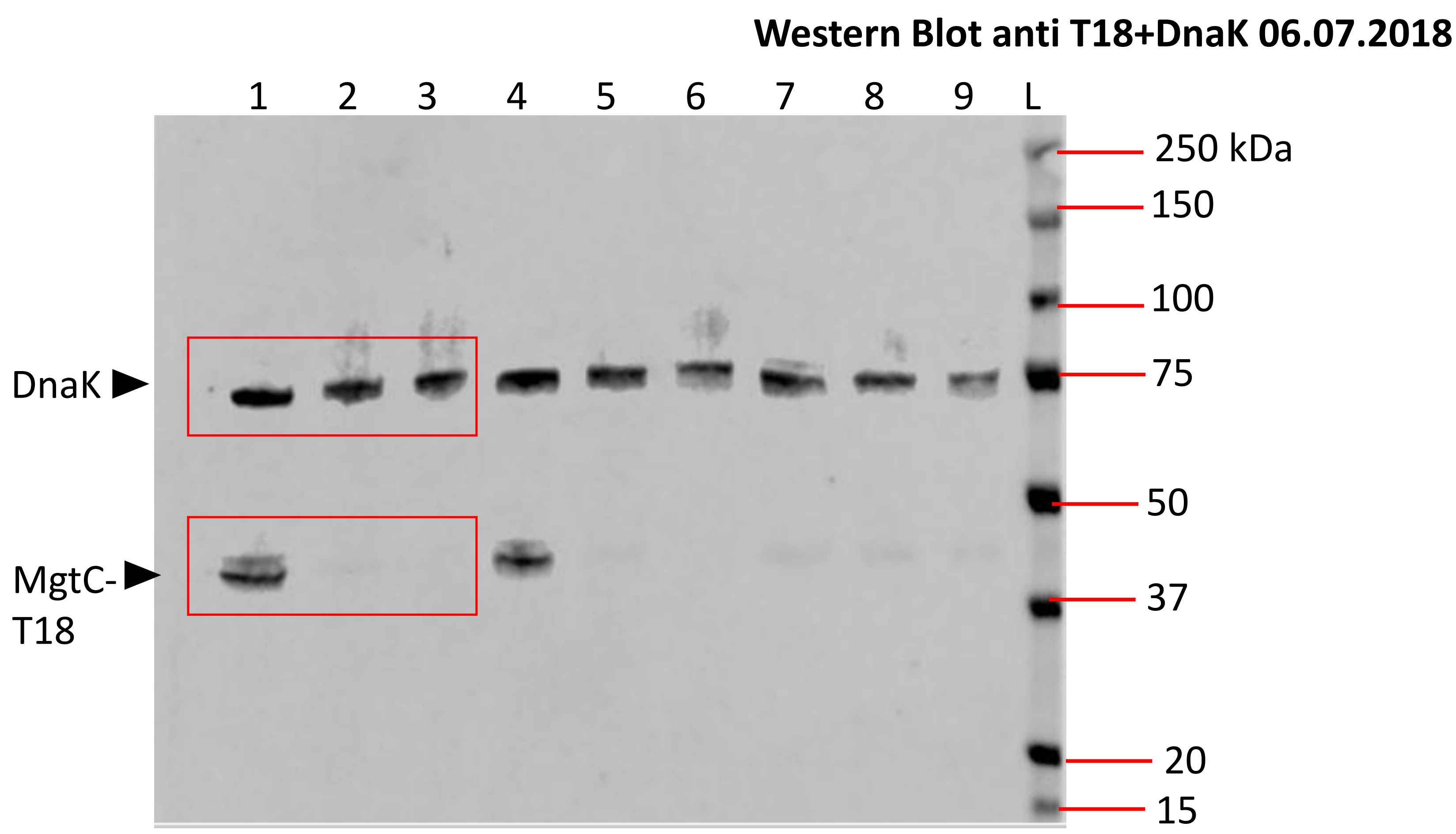

1 BTH101 pMgtC-T18 + DMSO
2 BTH101 pMgtC-T18 + MgtrS17I
3 BTH101 pMgtC-T18 + MgtrS17IC22S

Ladder

Figure 6C

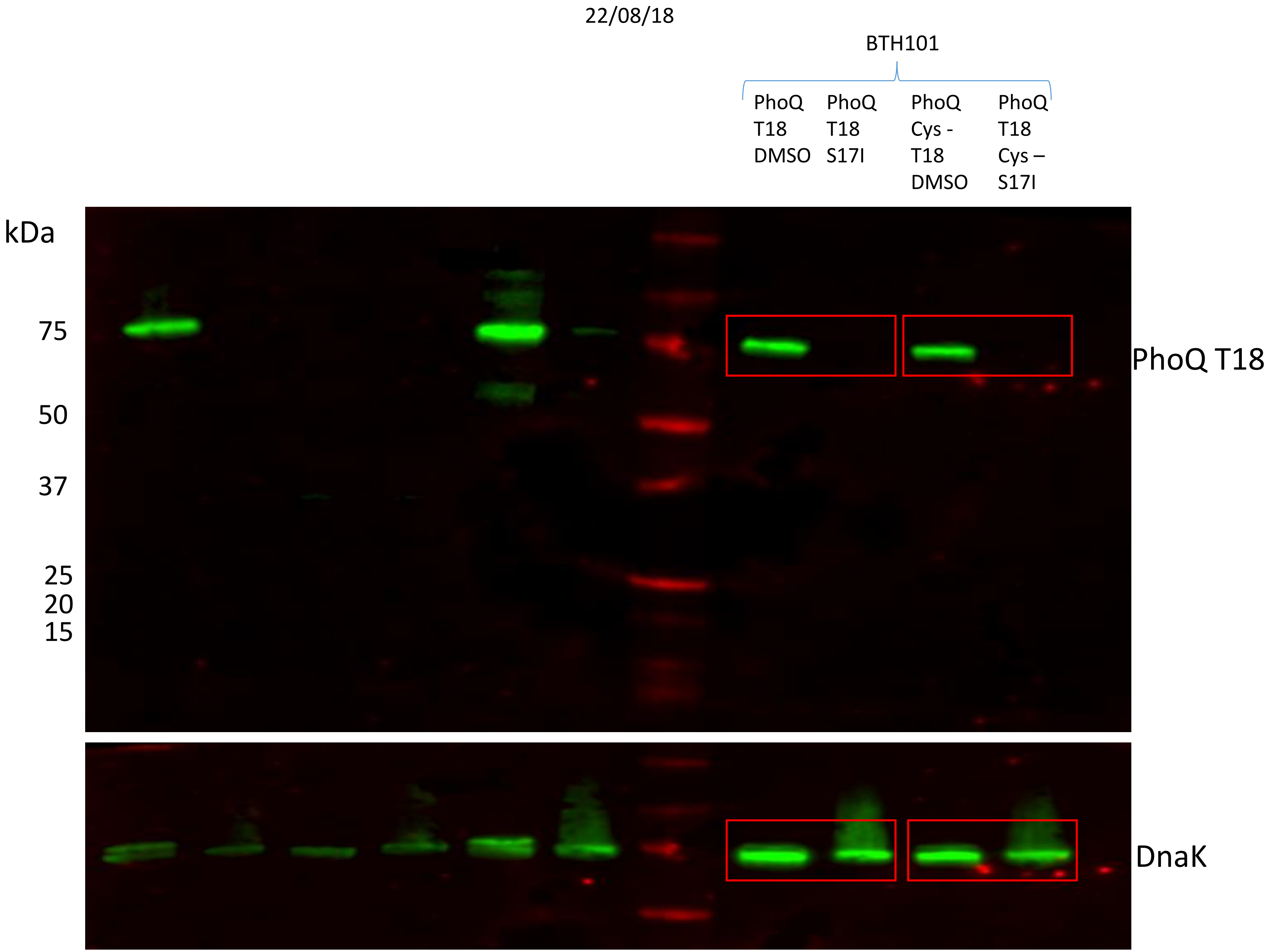

22/08/18
BTH101
PhoQ T18 DMSO
PhoQ T18 S17I
PhoQ Cys - T18 DMSO
PhoQ T18 Cys – S17I
kDa
75
50
37
25
20
15
PhoQ T18
DnaK

Figure 6D

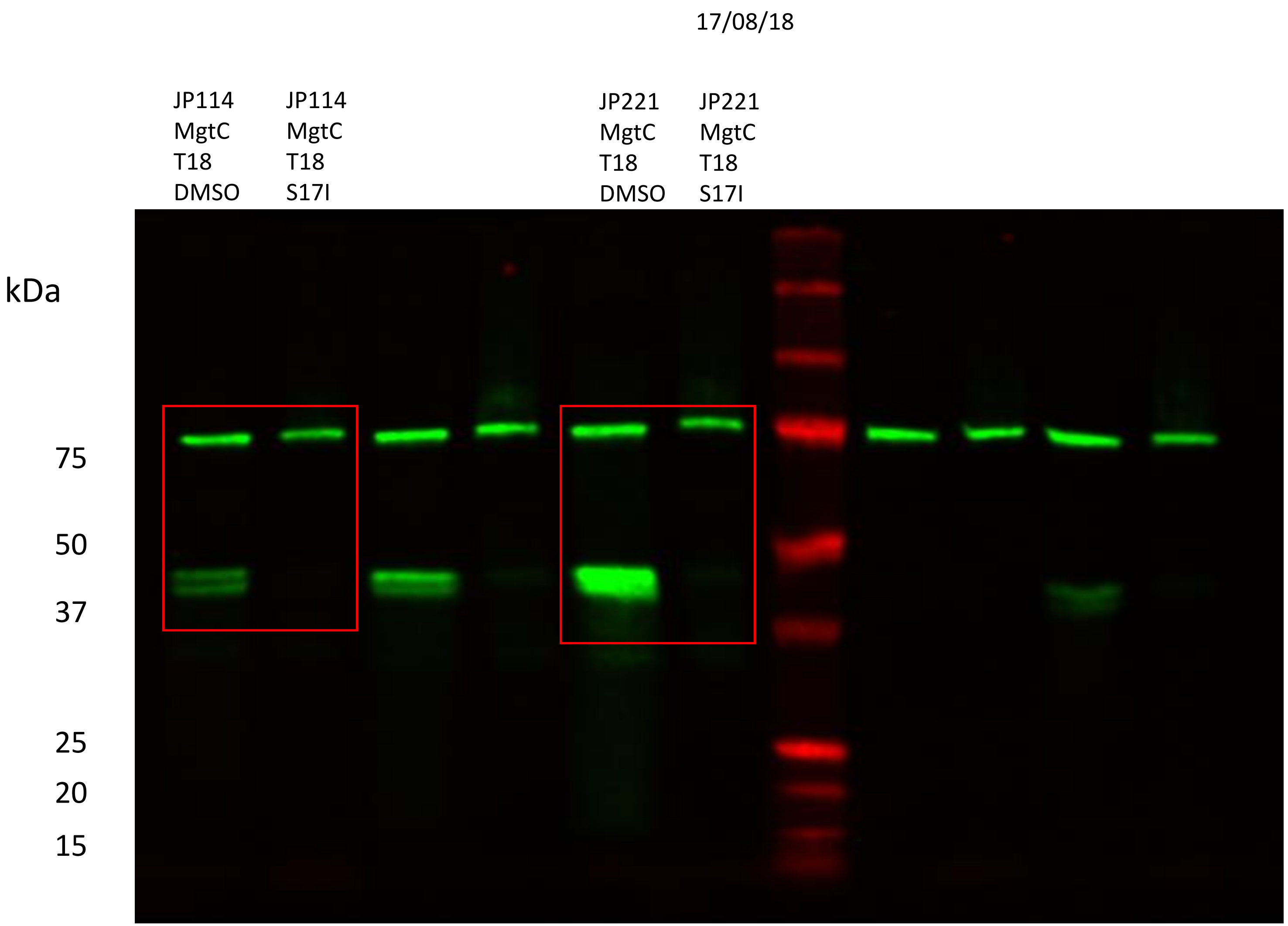
17/08/18
JP114 MgtC T18 DMSO
JP114 MgtC T18 S17I
JP221 MgtC T18 DMSO
JP221 MgtC T18 S17I
kDa
75
50
37
25
20
15

Figure 7C

WB BTH101 Test ScR S17I AcDnak AcT18 MgtC 03.04.19

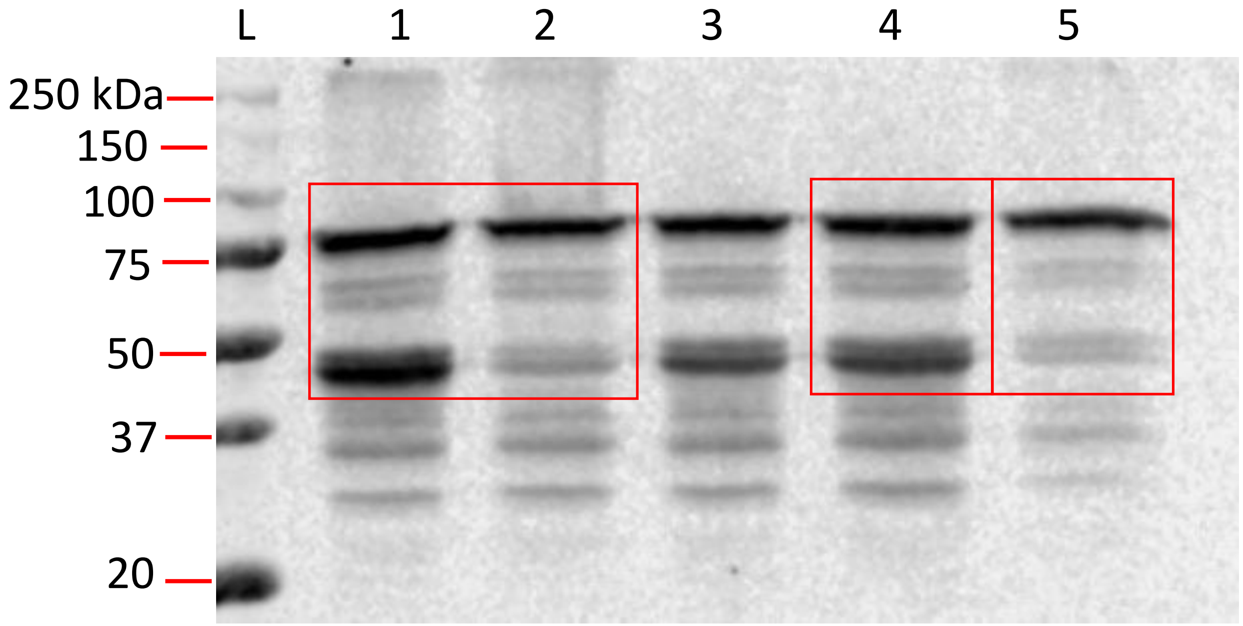

MgtC-T18

1 BTH101 pMgtC-T18 + DMSO

2 BTH101 pMgtC-T18 + MgtR

3 BTH101 pMgtC-T18 + Scr

4 BTH101 pMgtC-T18 + Scr

5 BTH101 pMgtC-T18 + MgtRS17I

Figure S2

24/05/18
SDS PAGE Pre-cast 4-12%

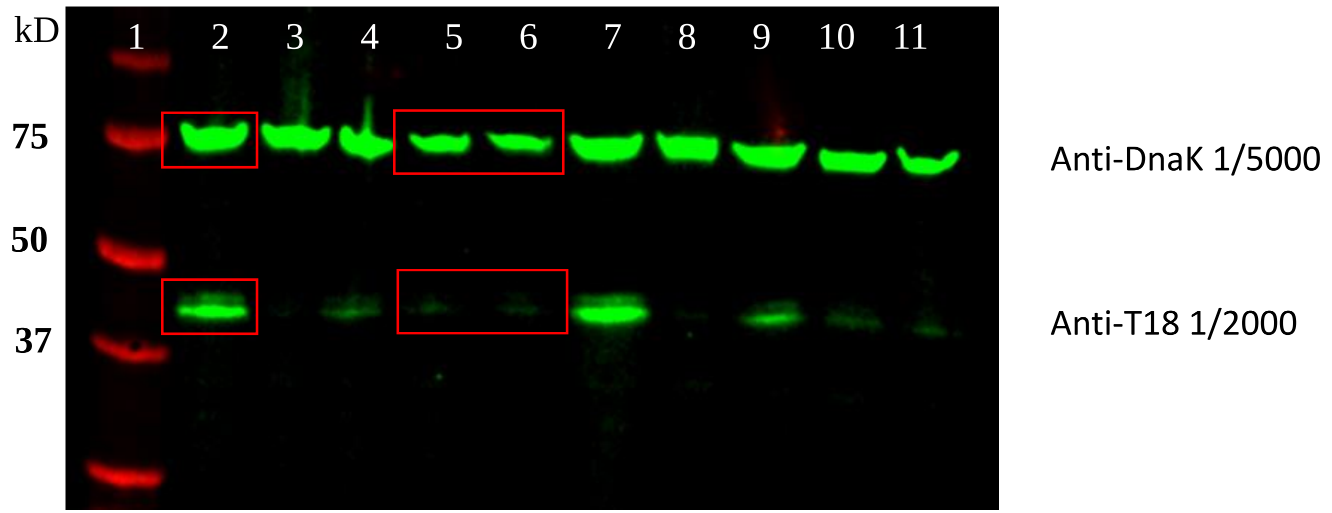

1 M
2 BTH101: MgtC St-18 DMSO
5 BTH101: MgtC St-18 S17I
6 BTH101: MgtC St-18 S17I short
