# Supplementary material for: Synthetic hydrophobic peptides derived from MgtR weaken Salmonella pathogenicity and work with a different mode of action than endogenously produced peptides
Source: Sci Rep. 2019 Oct 24;9:15253. doi: 10.1038/s41598-019-51760-2 (PMC6813294; doi:10.1038/s41598-019-51760-2)

**Synthetic hydrophobic peptides derived from MgtR weaken *Salmonella* pathogenicity and work with a different mode of action than endogenously produced peptides**

Mariana Rosas Olvera, Preeti Garai, Grégoire Mongin, Eric Vivès, Laila Gannoun-Zaki, Anne-Béatrice Blanc-Potard^1,2*^

**MgtR-WT**

**
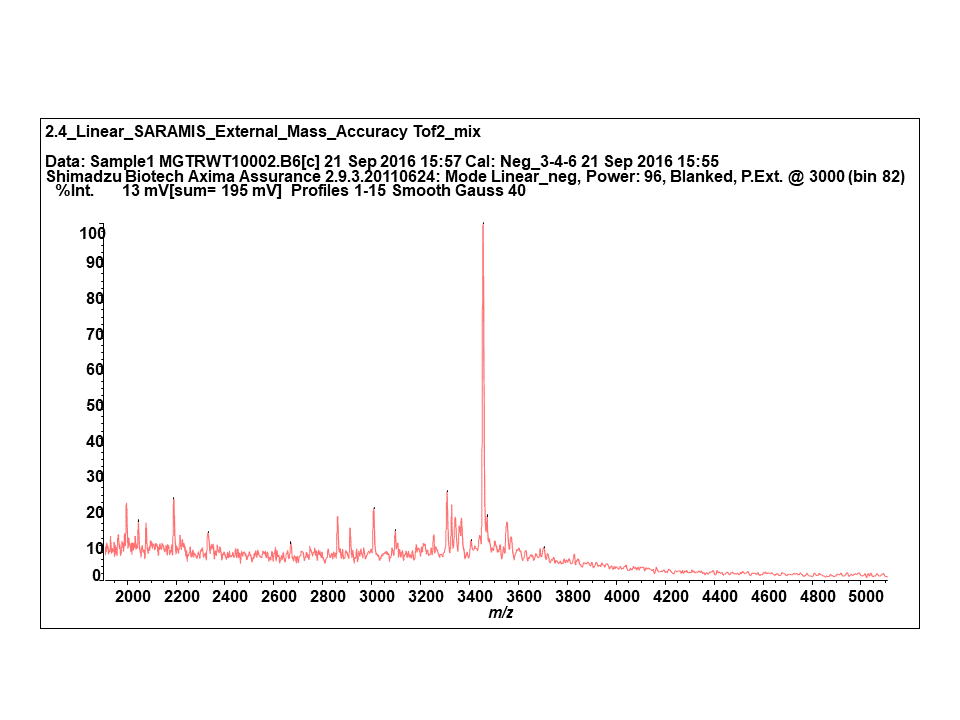
**

**MgtR-S17I**


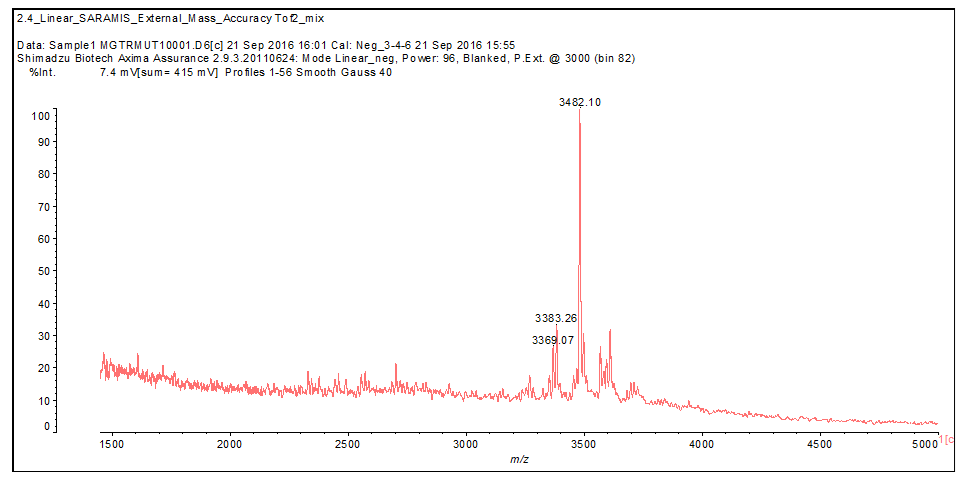


**MgtR-S17I-short**


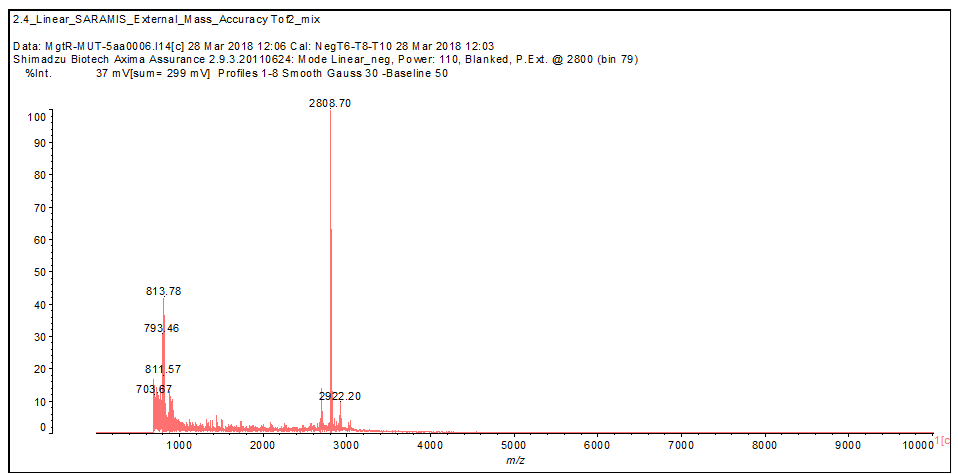


**MgtR-S17I-C22S**


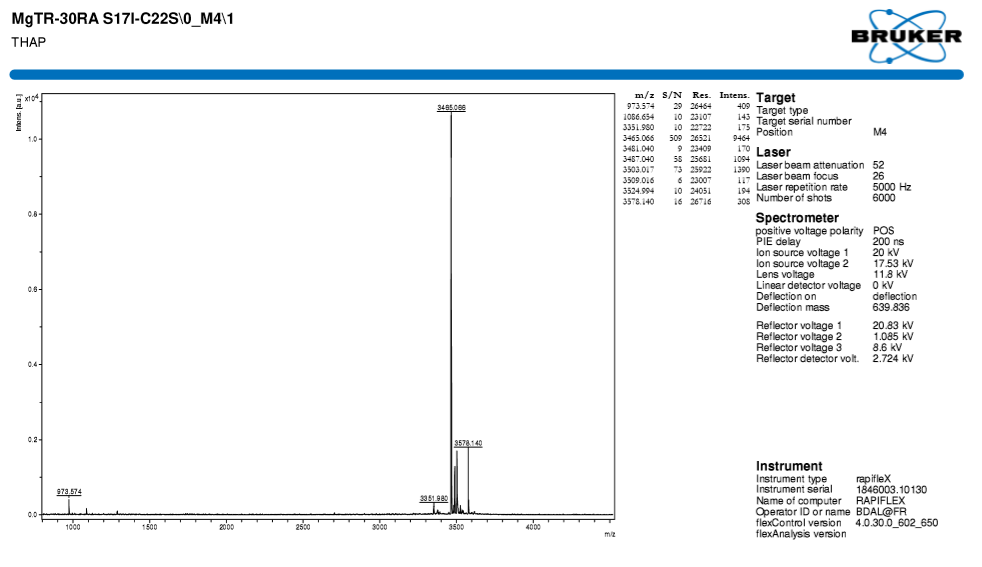


**MgtR-Scr**


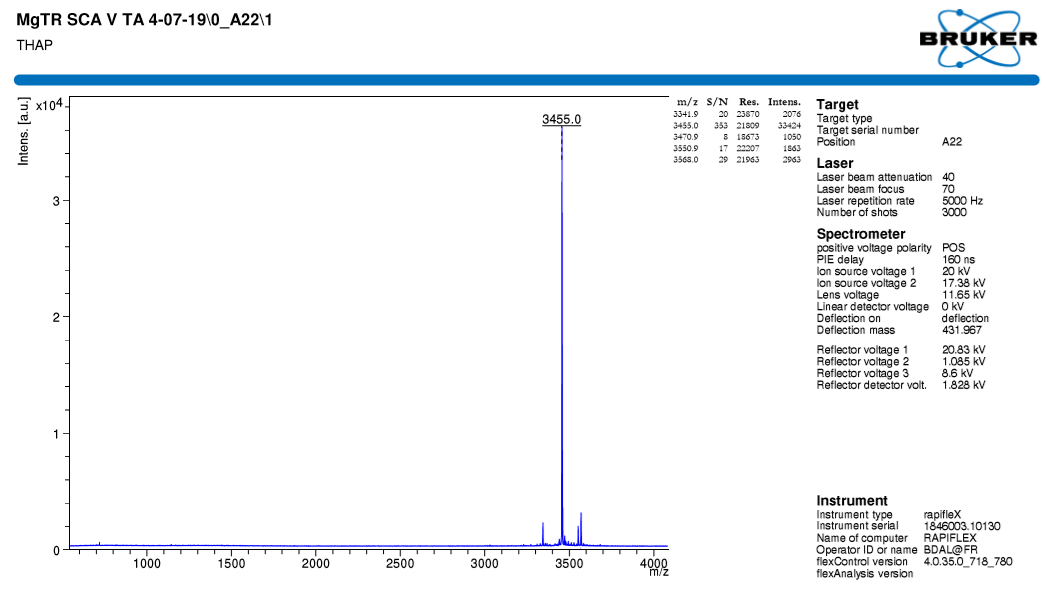


**Fluo-MgtR**


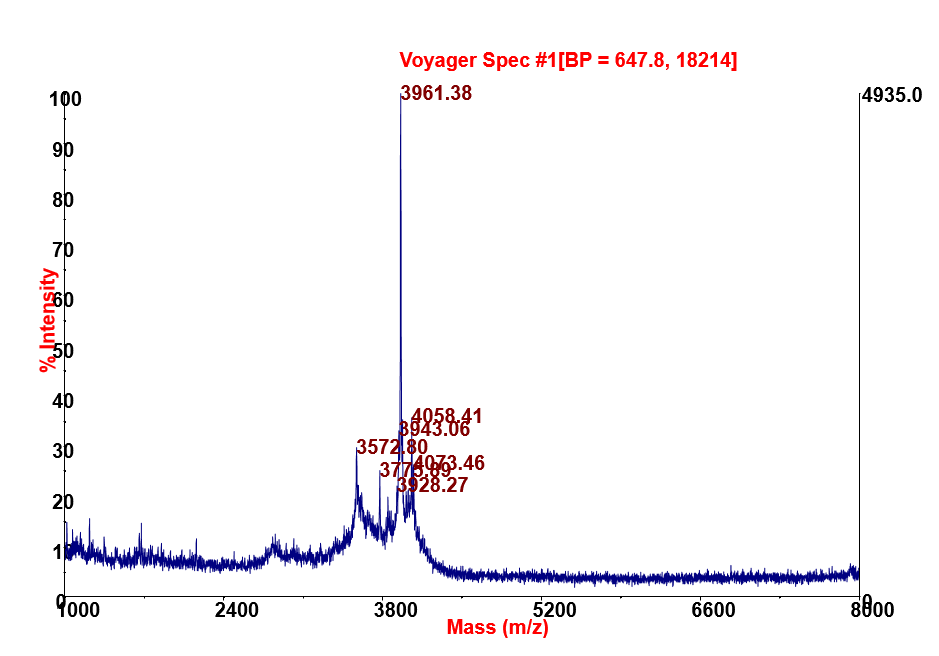


**MgtR-Fluo**


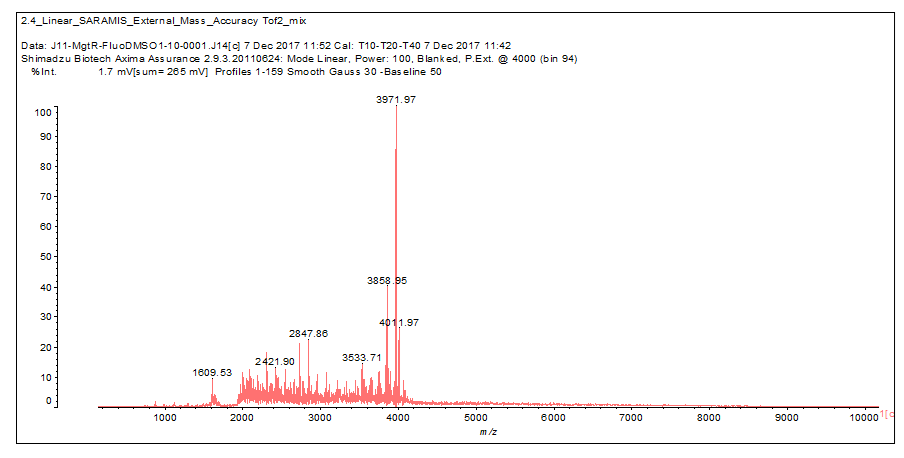

Supplement: Supplementary file 3 — Supplementary information for MALDI-TOF (full spectra) [file 41598_2019_51760_MOESM3_ESM.docx]
